# Trisomy of human chromosome 21 enhances amyloid-β deposition independently of an extra copy of *APP*

**DOI:** 10.1093/brain/awy159

**Published:** 2018-06-26

**Authors:** Frances K Wiseman, Laura J Pulford, Chris Barkus, Fan Liao, Erik Portelius, Robin Webb, Lucia Chávez-Gutiérrez, Karen Cleverley, Sue Noy, Olivia Sheppard, Toby Collins, Caroline Powell, Claire J Sarell, Matthew Rickman, Xun Choong, Justin L Tosh, Carlos Siganporia, Heather T Whittaker, Floy Stewart, Maria Szaruga, London Down syndrome consortium, Michael P Murphy, Kaj Blennow, Bart de Strooper, Henrik Zetterberg, David Bannerman, David M Holtzman, Victor L J Tybulewicz, Elizabeth M C Fisher, The LonDownS Consortium, Andre Strydom, Elizabeth Fisher, Dean Nizetic, John Hardy, Victor Tybulewicz, Annette Karmiloff-Smith

**Affiliations:** 1 Department of Neurodegenerative Disease, UCL Institute of Neurology, London, WC1N 3BG UK; 2 The LonDownS Consortium, Department of Forensic and Neurodevelopmental Sciences, Institute of Psychiatry, Psychology and Neuroscience, King’s College London, Denmark Hill, London, SE5 8AF, UK; 3 Department of Experimental Psychology, University of Oxford, Oxford, OX1 3PH, UK; 4 Department of Neurology, Washington University School of Medicine, St Louis, Missouri, 63110, USA; 5 Department of Psychiatry and Neurochemistry, Institute of Neuroscience and Physiology, University of Gothenburg, S-405 30, Sweden; 6 Sanders-Brown Center on Aging, University of Kentucky, Lexington, Kentucky, 40507, USA; 7 VIB-KU Leuven Center for Brain and Disease Research, VIB-Leuven 3000, Center for Human Genetics, Universitaire Ziekenhuizen and LIND, KU Leuven, Leuven, Belgium; 8 MRC Prion Unit at UCL, UCL Institute of Prion Diseases, 33 Cleveland Street, London W1W 7FF, UK; 9 Department of Molecular Neuroscience, UCL Institute of Neurology, London, WC1N 3BG, UK; 10 UK Dementia Research Institute, London, WC2B 4AN, UK; 11 Francis Crick Institute, London, NW1 1AT, UK; 12 Department of Medicine, Imperial College, London, SW7 2AZ, UK

**Keywords:** Down syndrome, Alzheimer’s disease, APP, amyloid-β, neurodegeneration

## Abstract

Down syndrome, caused by trisomy of chromosome 21, is the single most common risk factor for early-onset Alzheimer’s disease. Worldwide approximately 6 million people have Down syndrome, and all these individuals will develop the hallmark amyloid plaques and neurofibrillary tangles of Alzheimer’s disease by the age of 40 and the vast majority will go on to develop dementia. Triplication of *APP*, a gene on chromosome 21, is sufficient to cause early-onset Alzheimer’s disease in the absence of Down syndrome. However, whether triplication of other chromosome 21 genes influences disease pathogenesis in the context of Down syndrome is unclear. Here we show, in a mouse model, that triplication of chromosome 21 genes other than *APP* increases amyloid-β aggregation, deposition of amyloid-β plaques and worsens associated cognitive deficits. This indicates that triplication of chromosome 21 genes other than *APP* is likely to have an important role to play in Alzheimer’s disease pathogenesis in individuals who have Down syndrome. We go on to show that the effect of trisomy of chromosome 21 on amyloid-β aggregation correlates with an unexpected shift in soluble amyloid-β 40/42 ratio. This alteration in amyloid-β isoform ratio occurs independently of a change in the carboxypeptidase activity of the γ-secretase complex, which cleaves the peptide from APP, or the rate of extracellular clearance of amyloid-β. These new mechanistic insights into the role of triplication of genes on chromosome 21, other than *APP*, in the development of Alzheimer’s disease in individuals who have Down syndrome may have implications for the treatment of this common cause of neurodegeneration.

## Teaser

Wiseman *et al.* show that triplication of genes other than *APP* is sufficient to exacerbate Aβ deposition and associated cognitive changes in a mouse model of Down syndrome – Alzheimer’s disease. This occurs independently of changes to γ-secretase but results from a novel mechanism that lowers the soluble Aβ40/42 ratio.

## Introduction

Approximately 6 million individuals worldwide have Down syndrome. Changes in social attitudes and improvements in medical care have led to a significant rise in life expectancy for people who have Down syndrome (Glasson *et al.*, 2016). In particular, neonatal survival rates of individuals who have Down syndrome rose dramatically between 1950 and 1970 (Wu and Morris, 2013), such that today more people with the condition than ever before are approaching late middle age. Down syndrome is the biggest single genetic risk factor for Alzheimer’s disease (Wiseman *et al.*, 2015). A proportion of people with Down syndrome start to accumulate amyloid-β within their brain in childhood (Lemere *et al.*, 1996; Leverenz and Raskind, 1998), and the vast majority of individuals will have accumulated substantial amounts of amyloid-β by their mid-20s (Mann, 1988). By the age of 40, people who have Down syndrome will also have universally developed neurofibrillary tangles in a pattern broadly similar to that of Alzheimer’s disease in the general population (Mann, 1988; Lemere *et al.*, 1996; Leverenz and Raskind, 1998). The vast majority of people who have Down syndrome will develop early-onset Alzheimer’s disease dementia, for example, it is estimated that by their 60s, approximately two-thirds of individuals will have clinical dementia (McCarron *et al.*, 2014). The first site of amyloid-β accumulation in people with Down syndrome is within the cell (Gouras *et al.*, 2000; Gyure *et al.*, 2001; Mori *et al.*, 2002; Hirayama *et al.*, 2003) in the endo-lysosomal system (Cataldo *et al.*, 2000, 2004). It is clear that one of the earliest Alzheimer’s disease-associated pathological changes in people who have Down syndrome is the accumulation of amyloid-β. Understanding the genetic factors that influence amyloid-β accumulation in the context of trisomy of chromosome 21 may assist with the development of novel treatments for Alzheimer’s disease in this important population.

Duplication of *APP* alone, in the absence of chromosome 21 trisomy, is a cause of early onset Alzheimer’s disease (Rovelet-Lecrux *et al.*, 2006; Sleegers *et al.*, 2006) making it likely that three copies of *APP* are important in the development of Down syndrome–Alzheimer’s disease. However, the influence of three copies of the other >600 Hsa21 protein-coding and non-coding genetic elements (Ensembl GRCh38.p10) on Alzheimer’s disease in people who have Down syndrome is largely not understood.

Here, we report for the first time that triplication of chromosome 21 genes other than *APP*, increases amyloid-β aggregation, plaque formation, and cognitive deficits in a novel Down syndrome–Alzheimer’s disease (amyloid-β deposition) mouse model. In our unique model system, we show that trisomy of chromosome 21 lowers the ratio of soluble amyloid-β_40_ to amyloid-β_42_, a known pro-amyloidogenic change, and that this alteration in the peptide ratio correlates with amyloid-β aggregation in the brain. Importantly, we show that the effect of trisomy of chromosome 21 on soluble amyloid-β_40_/amyloid-β_42_ ratio occurs despite unaltered γ-secretase complex carboxypeptidase activity.

## Materials and methods

### Experimental design

For animal studies end-points were determined by animals’ age, as defined below (Cohorts A–I). All experiments were undertaken blind to genotype. Genotype was decoded after experimental analysis and reconfirmed using an independent DNA sample isolated from post-mortem tail, apart from instances of sudden death when tissue could not be recovered. Group sizes were calculated using power calculations, based upon estimates of error in the literature. Animals in which a reduction of copy number of the *APP* transgene was observed at initial genotyping at 3–4 weeks of age were excluded from further analysis. No outliers were excluded from the study. Individual animals were treated as the experimental unit.

Experiments using human post-mortem tissues were undertaken blind to euploid/trisomy status. Samples sizes were determined by the availability of tissues and appropriate matched controls at the suppling brain bank. Individual patients were treated as the experimental unit.

### Animal cohorts

Cohort A: (longitudinal behaviour and 16 months old for amyloid-β deposition studies) wild-type *n = *29, trisomic *n = *30, tgAPP *n = *19, trisomic;tgAPP *n = *17. Cohort B: (2–3-month-old behaviour and aged to 15 months) wild-type *n = *17, trisomic *n = *13, tgAPP *n = *17, trisomic;tgAPP *n = *9. Cohort C: (aged to 15 months, mice used in another study) wild-type *n = *14, trisomic *n = *11, tgAPP *n = *20, trisomic;tgAPP *n = *6. Cohort D: (6 months old for amyloid-β deposition and fractionation studies) wild-type *n = *12, trisomic *n = *12, tgAPP *n = *12, trisomic;tgAPP *n = *12. Cohort E: (2 months old for amyloid-β fractionation and multimeric amyloid-β) wild-type *n = *24, trisomic *n* = 22, tgAPP *n = *25, trisomic;tgAPP *n = *27. Cohort F: (3 months old for RNA, proteins, and biochemical activity assays) wild-type *n = *55, trisomic *n = *43, tgAPP *n = *53, trisomic;tgAPP *n = *43. Cohort G: (3 month old females for *in vivo* microdialysis) tgAPP *n = *8, trisomic;tgAPP *n = *6. Cohort H: (3 months old for γ-secretase enzymatic assays) wild-type *n = *10, trisomic *n = *10. Cohort I: (4.5–6.5 months old for immunohistochemistry APP and amyloid-β study) wild-type *n = *10, trisomic *n = *10, tgAPP *n = *12, trisomic;tgAPP *n = *10.

### Statistical analysis

Data were analysed as indicated in the figure legends by either two-tailed Students *t*-test (single variable study) or univariate ANOVA (to control for multiple variables). For ANOVA between-subject factors for mouse work were: tg*APP*, trisomy, and sex; for experiments using human post-mortem material: age at death, sex, post-mortem interval, and disease/syndrome status. Additional between-subjects factors were included in ANOVA as follows, for study of amyloid-β (82E1) and APP (22C11) staining on samples aged between 4.5 and 6.5 months, age in days was included and for study of Tris-soluble amyloid-β_38_, amyloid-β_40_ and amyloid-β_42_ preparation batch and age in days of mouse were included. Repeated measures ANOVA was used for technical replicates when every sample had the same number of replicates run, including immunohistology studies where two sections were stained from each mouse, plaque counts made by the two independent scorers, and technical replicates in the γ-secretase carboxypeptidase activity study. For cases when the number of technical replicates varied between subjects, subject means were calculated and used in the ANOVA. For the longitudinal analysis of T-maze alternation (short-term memory) performance at different ages, a repeated measures ANOVA was used [between-subject factors tg*APP*, trisomy, sex, batch of mice Cohort A(i) or Cohort A(ii); within-subject factor age]. A repeated measures ANOVA was used for analysis of habituation to the open field [factors tg*APP*, trisomy, sex, batch of mice Cohort A(i) or Cohort A(ii)]. Univariate ANOVA was used for the other behavioural tasks (factors tg*APP*, trisomy, sex, batch of mice) [Cohort A(i), Cohort A(ii), and/or Cohort B]. The identity (i.e. physical location) of ‘other arm’ used during the exposure phase was included as an additional between-subjects factor in the analysis of the spatial Y-maze data. All analyses were performed in SPSS.

### Human tissue ethics

The procurement and use of human tissues in this study was in accordance with the UK Human Tissue Act 2004. The study was reviewed and approved by NHS Research Ethics committee, London-Queen Square. All samples were supplied, anonymized by UK Brain Banks, as indicated in Supplementary Table 1, and had full research consent.

### Animal welfare and husbandry

Mice were housed in controlled conditions in accordance with guidance issued by the Medical Research Council in Responsibility in the Use of Animals for Bioscience (2017) and all experiments were carried out under License from the UK Home Office and with Local Ethical Review panel approval. Tc1 mice were taken from a colony maintained by mating Tc1 females (MGI: 3814712) to F1 (129S8 × C57BL/6) males. J20 B6.Cg-Tg(PDGFB-APPSwInd)20Lms/2J (MGI: 3057148) animals were maintained by mating J20 *APP* transgenic mice to C57BL/6J. Tc1 females were mated to J20 males to generate Cohorts A–J, all cohorts were mixed sex unless otherwise stated. All mice were co-housed throughout the study as lone-housing is known to modify APP-related phenotypes (Kang *et al.*, 2007); mice were housed with littermates and/or animals of the same sex weaned at the same time, thus mice of differing genotypes were co-housed pseudo-randomly.

Mice had access to a mouse house with bedding material and wood chips. All animals had continual access to water and RM1 (Special Diet Services) (stock animals) or RM3 (Special Diet Services) (breeding animals) chow. Mice in Cohorts B–F and H–J were housed in individually ventilated cages in a specific pathogen free (SPF) facility. Mice in Cohorts A and G were bred in individually ventilated cages in an SPF facility prior to transportation to another facility. Mice from Cohort A were then housed in open cages in a non-SPF facility. Mice in Cohorts A–F and H–J were housed at 21 ± 2°C and 55 ± 10% humidity. Animals were euthanized by exposure to rising carbon dioxide, followed by confirmation of death by dislocation of the neck in accordance with the Animals (Scientific Procedures) Act 1986 (United Kingdom).

### DNA extraction and genotyping

DNA was extracted from tail tip or ear biopsy by the Hot Shot method (Truett *et al.*, 2000). Mice were genotyped using polymerase chain reaction (PCR) for the presence of human chromosome 21 (Tc1 specific primers f: 5′-GGTTTGAGGGAACACAAAGCTTAACTCCCA-3′ r: 5′-ACAGAGCTACAGCCTCTGACACTATGAACT-3′, control primers f: 5′-TTACGTCCATCGTGGACAGCAT-3′ r: 5′-TGGGCTGGGTGTTAGTCTTAT-3′) as described previously (O’Doherty *et al.*, 2005). Presence of the human *APP* transgene was tested by PCR using primers (APP f: 5′-GGTGAGTTTGTAAGTGATGCC-3′ r: 5′-TCTTCTTCTTCCACCTCAGC-3′, control primers f: 5′-CAAATGTTGCTTGTCTGGTG-3′ r: 5′-GTCAGTCGAGTGCACAGTTT-3′). Relative copy number of the human *APP* transgene was checked by quantitative PCR using a TaqMan™ Fast (ABI) (human *APP* transgene primers f: ‘5′-TGGGTTCAAACAAAGGTGCAA-3′ r: 5′-GATGAAGATCACTGTCGCTATGAC-3′ probe FAM-CATTGGACTCATGGTGGGCGGTG-3′ control primers f: 5′-CACGTGGGCTCCAGCATT-3′ r: 5′-TCACCAGTCATTTCTGCCTTTG-3′ control probe VIC-CCAATGGTCGGGCACTGCTCAA-3′.

### RNA extraction and quantitative RT-PCR

Total hippocampal RNA was extracted using miRNeasy® mini kit (Qiagen). Tissue was disrupted using a TissueRuptor® (Qiagen), and the protocol followed as per the manufacturer’s instructions, samples were defrosted and homogenized on ice. Final extracted RNA was eluted in DNase- and RNase-free water. Amounts of RNA were equalized and cDNA was generated using the QuantiTect® Reverse Transcription Kit (Qiagen). Quantitative PCR was undertaken to determine expression of human mutant *APP* (primers f: 5′-CGACCGAGGACTGACCACTC-3′ r: 5′-TGTCGGAATTCTGCATCCAGA-3′ probe FAM-CCAGGTTCTGGGTTGACAAATATCAAGACG) and mouse App (primers f: 5′-CTCCAGCCGTGGCACC-3′ r: 5′-AGTCCTCGGTCAGCAGCG-3′ probe FAM-ACTCTGTGCCAGCCAATACCGAAAATGA). Mouse β-actin (*Actb*) (4352341E-Vic Life Technologies) and *Gapdh* (4342339E-Vic Life Technologies) were used as endogenous controls. Minus reverse-transcriptase controls were run for every sample for all reactions. No evidence of genomic amplification was detected.

### Tissue preparation and western blotting

For analysis of protein abundance in hippocampus and cortex, tissue was dissected under ice-cold PBS before snap freezing. Samples were then homogenized in RIPA Buffer (150 mM sodium chloride, 50 mM Tris, 1% NP-40, 0.5% sodium deoxycholate, 0.1% sodium dodecyl sulphate) plus complete protease inhibitors (Calbiochem) by mechanical disruption. Total protein content was determined by Bradford assay. Samples from individual animals were run separately and were not pooled.

Equal amounts of total brain proteins were then denatured in LDS denaturing buffer (Invitrogen) and β-mercaptoethanol, prior to separation by SDS-PAGE gel electrophoresis using precast 4–12% Bis-Tris gels (Invitrogen). Proteins were transferred to nitrocellulose or PVDF membranes prior to blocking in 5% milk/PBST (0.05% Tween-20) or 5–10% bovine serum albumin (BSA)/PBST. Primary antibodies were diluted in 1% BSA/PBST, HRP-conjugated secondary anti-rabbit, anti-mouse and anti-goat antibodies (Dako) were diluted 1:10 000 in 1% BSA/PBST. Linearity of antibody binding was confirmed by a 2-fold dilution series of cortical protein samples. Band density was analysed using ImageJ. Relative signal of the antibody of interest compared to the internal loading control was then calculated, and relative signal was then normalized to mean relative signal of control samples run on the same gel. Mean of technical replicates were calculated and used for ANOVA, such that biological replicates were used as the experimental unit.

Primary antibodies against C-terminal APP (Sigma A8717, 1:10 000), BACE1 (Abcam ab108394, 1:1000), IDE (Abcam ab32216, 1:1000), Neprilysin (R&D Systems AF1126, 1:1000), β-actin (Sigma A5441, 1:60 000), and GAPDH (Sigma G9545, 1:200 000), were used.

### BACE1 β-secretase activity assay

BACE1 β-secretase activity was measured as described previously (Ahmed *et al.*, 2010). Briefly, whole cortex was lysed in extraction buffer (10 mM sodium acetate, 3 mM NaCl, 0.1% Triton™ X-100, 0.32 M sucrose, pH 5.0) and an anti-BACE1 antibody (Abcam 108394) was used to capture endogenous BACE1, and cleavage of a β-secretase fluorogenic peptide substrate was measured over 2 h at 37°C.

### Carboxypeptidiase γ-secretase activity assay

CHAPSO detergent resistant membranes (DRMs) were prepared from brain cortices after careful removal of leptomeninges and blood vessels, as previously described (Szaruga *et al.*, 2015). Briefly, tissue was homogenized in ∼10 volumes of 10% sucrose in MBS buffer (25 mM MES, pH 6.5, 150 mM NaCl) containing 1% CHAPSO (Sigma); separated by a sucrose density gradient and the DRM fraction (interface of 5%/35% sucrose) was collected and rinsed twice in 20 mM PIPES, pH 7, 250 mM sucrose, 1 M EGTA. The resultant pellet was resuspended with the above buffer and used as source of enzyme. Activity assays were carried out for 1 or 2 h for mouse or human derived DRMs, respectively, as described before (Szaruga *et al.*, 2015).

### Expression and purification of wild-type C99-3xFLAG substrate

Human wild-type APPC99-3xFLAG substrate was expressed in COS1 or HEK cells and purified as previously described (Chavez-Gutierrez *et al.*, 2008). Purity was assessed by SDS-PAGE and Coomassie staining (GelCode reagent, Pierce).

### Quantification of amyloid-β production by ELISA

Amyloid-β_38_, amyloid-β_40_ and amyloid-β_42_ product levels were quantified on Multi-Spot 96 well plates pre-coated with anti-amyloid-β_38_, amyloid-β_40_, and amyloid-β_42_ antibodies obtained from Janssen Pharmaceutica using multiplex MSD technology, as described before (Szaruga *et al.*, 2015).

### Tissue fractionation for amyloid-β or soluble APP assays and amyloid-β ELISA

Total cortical proteins were fractionated based on the method in Shankar *et al.* (2009). Total cortex was homogenized in five volumes of ice-cold Tris-buffered saline (TBS) (50 mM Tris-HCl pH 8.0) plus complete protease and phosphatase inhibitors (Calbiochem). Homogenates were centrifuged at 175 000 *g* at 4°C for 30 min, and the resultant supernatant (the soluble TBS fraction) was stored at −80°C. The resultant pellet was homogenized in five volumes of 1% Triton™ X-100 in TBS plus complete protease inhibitors and centrifuged at 175 000 *g* at 4°C for 30 min, and the resultant supernatant (the Triton soluble fraction) was stored at −80°C. The resultant pellet was homogenized in eight volumes of 50 mM Tris-HCl buffer, pH 8.0, containing 5 M guanidine-HCl plus complete protease inhibitors (Calbiochem). This resuspension (the guanidine HCl soluble fraction) was incubated at 4°C for a minimum of 14 h with shaking and was stored at −80°C. Protein concentration was determined by Bradford assay (Bio-Rad).

Total hippocampal proteins were homogenized in five volumes of ice-cold TBS plus complete protease and phosphatase inhibitors (Calbiochem) based on the method in Holtta *et al.* (2013). Homogenates were centrifuged at 16 000 *g* at 4°C for 30 min. The resultant supernatant (the soluble TBS fraction) was stored at −80°C. Protein concentration was determined by Bradford assay (Bio-Rad).

Samples were then analysed by human amyloid-β_40_ and amyloid-β_42_ ELISA (Life Technologies), and/or amyloid-β 6E10 Triplex, sAPPβ or sAPPα Assay (Meso Scale Discovery) following the manufacturer’s protocols. Briefly the TBS, Triton, and guanidine HCl soluble fractions were diluted into reaction buffer (Dulbecco’s phosphor-buffered saline 5% BSA 0.0003% Tween, complete protease inhibitors) or Diluent 35 (Meso Scale Discovery) and added to a precoated plate prior to addition of amyloid-β detection antibody and incubation overnight at 4°C (Invitrogen) or 2 h at room temperature (Meso Scale Discovery). After washing, either a HRP-secondary antibody (Life Technologies) was applied prior to application of a chromogenic reagent (Life Technologies) and plates were read on a Sunrise plate-reader (450 nm), or Read Buffer (Meso Scale Discovery) was applied immediately prior to plate reading on a Meso Scale Discovery Sector Imager.

### Multimeric amyloid-β ELISA

Multimeric amyloid-β was measured as described before in detail (Holtta *et al.*, 2013). In brief, the amyloid-β N-terminal-specific antibody 82E1 (IBL International) was used for both capture and detection, which results in selective quantification of oligomerized amyloid-β (no signal from monomers due to epitope-blocking). A synthetic dimer consisting of two amyloid-β_1–11_ peptides with an added C-terminal cysteine through which the peptides were coupled via a disulphide bridge (Caslo) was used to create the standard curve. All samples were measured on one occasion using one batch of reagents. The intra-assay coefficient of variation was 7%.

### Mass-spectrometry analysis of amyloid-β in fractionated cortical proteins

Amyloid-β was immunoprecipitated using the KingFisher magnetic particle processor (Thermo Fisher Scientific) and mass spectrometric analysis using MALDI-TOF MS were performed as described previously (Mustafiz *et al.*, 2011). Briefly, anti-amyloid-β antibodies 6E10 and 4G8 (Signet Laboratories) were separately added to magnetic Dynabeads® M-280 Sheep Anti-Mouse IgG (Invitrogen). These coated beads were mixed and added to fractionated brain homogenate diluted in 0.025% Tween 20 PBS (pH 7.4). After washing, using the KingFisher magnetic particle processor, bound amyloid-β was eluted using 0.5% formic acid. MALDI-TOF MS measurements were performed using an autoflex™ instrument (Bruker Daltonics). Each spectrum represents an average of 10 000 measurements. The MALDI samples were prepared with the seed layer method using cyano-4-hydroxycinnamic acid as the matrix. The area of each form of amyloid-β in the spectra was normalized to the sum of all areas of the amyloid-β peptides detected in the spectra, such that relative changes in the abundance of the different amyloid-β peptides can be calculated. It should be noted that the ratio between the different isoforms detected in the mass spectrum cannot be interpreted as a direct reflection of their absolute abundance in the brain since the ionization efficiency might be different for the different peptides and as different peptides are more hydrophobic and less soluble than others.

### *In vivo* microdialysis

*In vivo* microdialysis was used to assess brain interstitial fluid (interstitial fluid) amyloid-β_40_ half-life in awake mice as previously described (Castellano *et al.*, 2011). Briefly, unilateral guide cannula were implanted into the hippocampus and used to insert a 2 mm microdialysis probe (38 kDa molecular weight cut-off, BR-2, Bioanalytical Systems). Artificial CSF containing 0.15% BSA was used as perfusion buffer. Hourly interstitial fluid samples were collected for 6 h, using a 1 μl/min flow rate to determine basal level of interstitial fluid amyloid-β, prior to the administration of compound E by intraperitoneal injection (20 mg/kg body weight). After compound E administration, which is a γ-secretase inhibitor that prevents further amyloid-β production, samples were collected hourly for a further 6 h. Levels of amyloid-β_40_ were determined for each time-point using a sandwich ELISA (anti-amyloid-β_35–40_ HJ2 capture antibody and anti-amyloid-β_13–18_ HJ5.1-biotin as detecting antibody). The elimination of amyloid-β from the interstitial fluid followed first-order kinetics; therefore, for each mouse, t1/2 for amyloid-β was calculated with the slope, k′, of the linear regression that included all fractions until the concentration of amyloid-β stopped decreasing (t1/2 = 0.693/k, where k = 2.303k′; Castellano *et al.*, 2011).

### Immunohistochemistry of mouse brain

The brains were immersion fixed in 10% buffered formal saline (Pioneer Research Chemicals) for a minimum of 48 h prior to being processed to wax (Leica ASP300S tissue processor). The blocks were trimmed laterally from the midline by ∼0.9–1.4 mm to give a sagittal section of the hippocampal formation. Two 4 μm sections 40-μm apart were analysed. The sections were pretreated with 98% formic acid for 8 min, then antigen retrieval was undertaken by incubation for 30 min in Tris boric acid EDTA buffer (pH 9.0). Slides were then blocked prior to the application of directly biotinylated mouse monoclonal IgG1 antibodies against either full-length APP (22C11, MAB348B, Millipore, 1:3000), or amyloid-β (82E1, IBL, 0.2 μg/ml) for 8 h. This was followed by treatment with the Ventana DABMap™ kit (iView DAB, Ventana Medical Systems) using a Ventana XT automated stainer (Ventana Medical Systems). Alternatively, for staining of amyloid-β, slides were incubated with mouse monoclonal 6F/3D (Dako 1:50) followed by iVIEW™ Ig secondary antibody (Ventana Medical Systems). The sections were counterstained with haematoxylin, scanned (Leica SCN400F scanner) and analysed using Definiens software. 6F/3D stained slides were photographed (ImageView II 3.5 Mpix digital camera) and composed with Adobe Photoshop so that the entire cortex could be analysed. The same thresholds for staining intensity were then used to quantify the area covered by DAB stain using Volocity image analysis software (Perkin Elmer). Plaque numbers were counted by two independent scorers, using the counting objects feature of ImageJ.

### Behavioural study design

Cohort A was longitudinally tested in the following order: test 1, spontaneous alternation in the T-maze at 2–3 months of age; test 2, spontaneous alternation in the T-maze at 6–7 months of age; test 3, spatial novelty preference in the Y-maze at 6–7 months of age; test 4, habituation to an open field at 6–7 months of age; test 5, spontaneous alternation in the T-maze at 15–16 months of age. Cohort A was batched into two groups to facilitate testing (batch i and ii). Cohort B was tested at 2–3 months of age in the spatial novelty preference Y-maze task only, before being aged to 15 months. All behavioural testing was undertaken between 8.30 and 16.30 h (light 7.00–19.00 and dark 19.00–7.00). Cohorts A and B were tested at independent facilities, such that site of test compounded analysis of effect of age in the Y-maze task.

### Spontaneous alternation in the T-maze

A T-shaped maze made of wood painted dark grey with 30 × 10 × 29 cm arms, with a central partition extending 7 cm into the start arm from the back of the maze, was used to assess spontaneous alternation as previously described (Deacon and Rawlins, 2006). Mice were placed into the maze facing the wall of the start arm and allowed to make a free choice of either goal arm. The mouse was then restricted to that goal arm for 30 s by use of a guillotine door. The central partition was then removed and all doors reopened. The mouse was again placed at the end of the start arm facing the wall and allowed to make a further free choice of either goal arm. This was performed twice a day, once prior to 12.30 and once after 12.30 for 8 days, with an approximate interval of 4 h between the two daily sessions. Whether or not the animal chose the novel arm on the second run was recorded and summed across 16 trials.

### Spatial novelty preference Y-maze task

Spatial novelty preference was assessed in an enclosed Perspex® Y-maze as described previously (Sanderson *et al.*, 2007). Briefly, a Perspex® Y-maze with arms of 30 × 8 × 20 cm was placed into a room containing a variety of extra-maze cues. Mice were assigned two arms (the ‘start’ and the ‘other’ arm) to which they were exposed during the first phase (the exposure phase), for 5 min. This selection of arms was counterbalanced with respect to genotype. Timing of the 5-min period began only once the mouse had left the start arm. The mouse was then removed from the maze and returned to its home cage for a 1-min interval between the exposure and test phases. During the test phase, mice were allowed free access to all three arms. Mice were placed at the end of the start arm and allowed to explore all three arms for 2 min beginning once they had left the start arm. An entry into an arm was defined by a mouse placing all four paws inside the arm. Similarly, a mouse was considered to have left an arm if all four paws were placed outside the arm. The times that mice spent in each arm were recorded manually and a novelty preference ratio was calculated for the time spent in arms [novel arm / (novel + other arm)].

### Habituation to an open field

Mice were habituated to a grey arena (40 × 40 × 40 cm) under low light levels (20 lx), for 10 min/day on three consecutive days [at a similar time each day (±30 min)]. Distance travelled was measured using EthoVision. The ‘outer’ zone was defined as 10 cm from the edge of the box and the inner zone as the 20 cm^2^ area in the centre.

## Results

### Trisomy of chromosome 21 genes other than *APP* promotes the deposition of amyloid-β

Having three copies of chromosome 21 genes other than *APP* may influence the development of Alzheimer’s disease in people who have Down syndrome. This is partly based on mouse studies using single gene overexpression models, rather than animal models that are aneuploid (Wiseman *et al.*, 2015). To take an unbiased approach and investigate if triplication of genes other than *APP* can modulate the development of Down syndrome–Alzheimer’s disease *in vivo*, we crossed a model of Down syndrome that is aneuploid for chromosome 21 with a mouse that deposits amyloid-β in the brain. Thus, we have generated a new model system to understand the early stages of Down syndrome–Alzheimer’s disease, when amyloid-β starts to accumulate.

The Down syndrome mouse was the Tc(Hsa21)1TybEmcf (Tc1) model (O’Doherty *et al.*, 2005), which carries a freely segregating chromosome 21 and is trisomic for 75% of the genes on this chromosome but, importantly, is not functionally triplicated for *APP* (Sheppard *et al.*, 2012; Gribble *et al.*, 2013). As with a small percentage of Down syndrome individuals, the mouse is mosaic; on average ∼66% of brain nuclei (neurons and glia) retain chromosome 21 (O’Doherty *et al.*, 2005). This model has well-defined Down syndrome-associated deficits, including defects in nervous system function such as in long-term potentiation, short-term memory, dendritic spine morphology and connectivity in the hippocampus (O’Doherty *et al.*, 2005; Morice *et al.*, 2008; Witton *et al.*, 2015; Hall *et al.*, 2016).

To determine if trisomy of chromosome 21 sequences other than *APP* is sufficient to modify Alzheimer’s disease-related phenotypes, we crossed Tc1 mice with the *APP* transgenic mouse strain Tg(PDGFB-APPSwInd)20Lms (J20-tgAPP). J20-tgAPP mice overexpress a human *APP* transgene with Alzheimer’s disease mutations and accumulates amyloid-β within the brain from ∼4–5 months of age (Mucke *et al.*, 2000). This model has been widely used to study amyloid deposition. Our ‘trisomic × tgAPP’ (Tc1 × J20-tgAPP) cross is an *in vivo* model system that allows us to investigate if amyloid deposition (from the tgAPP transgene) is modified in the presence of an additional copy of human chromosome 21, by producing four types of progeny: (i) wild-types; (ii) those inheriting the *APP* transgene only (‘tgAPP’); (iii) those inheriting human chromosome 21 only (‘trisomic’); and (iv) those that inherit both the *APP* transgene and human chromosome 21 (‘trisomic;tgAPP’).

**Figure 1:**
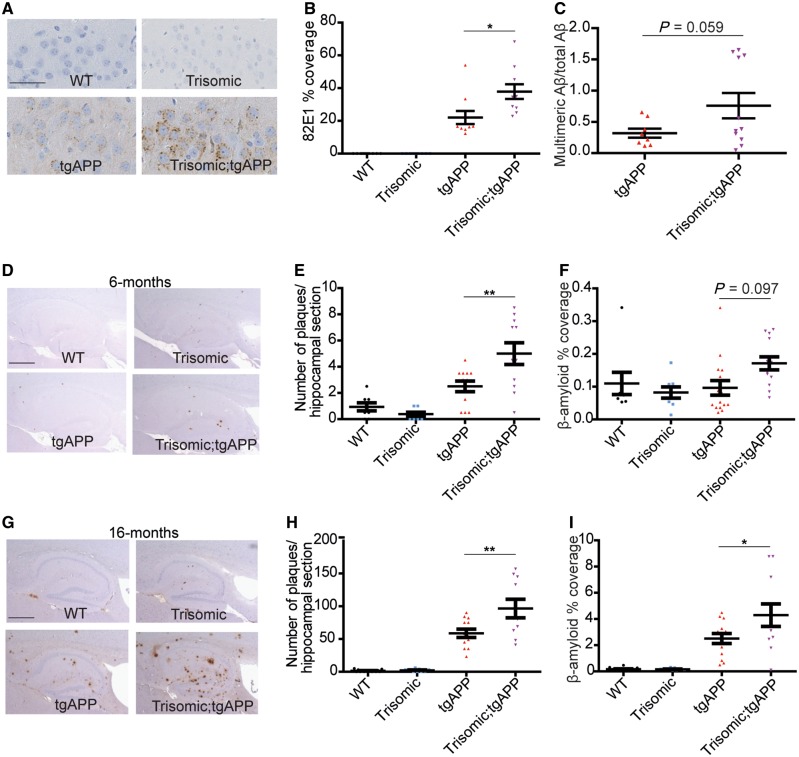
**Trisomy of chromosome 21 promotes the intracellular and extracellular deposition of amyloid-β.** (**A** and **B**) Intracellular amyloid-β deposition (82E1) was increased by trisomy in hippocampal CA3 pyramidal neurons in 4.5–6.5-month-old mice [ANOVA trisomy-tgAPP interaction *F*(1,31) = 5.125, *P = *0.031] [Bonferroni pairwise comparisons trisomic;tgAPP with tgAPP *P = *0.012; wild-type (WT) (black circles) *n = *10, trisomic (blue squares) *n = *10, tgAPP (red triangles) *n = *10, trisomic;tgAPP (purple inverted triangles) *n = *10]. (**C**) A trend for increased hippocampal Tris-soluble multimeric amyloid-β (82E1-82E1 ELISA) normalized to the sum of amyloid-β_38_, amyloid-β_40_ and amyloid-β_42_ in trisomic;tgAPP was observed [trisomy *F*(1,24) = 3.928, *P = *0.059; tgAPP *n = *8, trisomic;tgAPP *n = *11)]. (**D**–**I**) Amyloid-β deposition (6F/3D) in the hippocampus was quantified at (**D**–**F**) 6 and (**G**–**I**) 16 months of age. Trisomy increases (**E** and **H**) the number of plaques [tgAPP–trisomy interaction *F*(1,77) = 6.744, *P = *0.011, Bonferroni pairwise comparisons trisomic;tgAPP with tgAPP 6 months *P = *0.008, 16 months *P = *0.003] and (**F** and **I**) the area covered by amyloid-β [tgAPP–trisomy interaction *F*(1,85) = 4.005, *P = *0.049, Bonferroni pairwise comparisons trisomic;tgAPP with tgAPP 6 months *P = *0.097, 16 months *P = *0.037] (6 months wild-type *n = *8, trisomic = 8, tgAPP *n = *15, trisomic;tgAPP *n = *13; 16 months wild-type *n = *16, trisomic = 9, tgAPP *n = *13, trisomic;tgAPP *n = *11). Data are represented as mean ± SEM, *P < 0.05, **P < 0.01, ***P < 0.001. Both male and female mice were studied and sex was included as a factor in the ANOVA. Scale bar in **A** = 50 µm; **D** and **G** = 500 µm. Aβ = amyloid-β.

We observed that early intracellular amyloid-β deposition within the CA3 pyramidal cells of the hippocampus was significantly enhanced by trisomy of chromosome 21 at 4.5–6.5 months of age in trisomic;tgAPP mice compared to tgAPP littermates (Fig. 1A and B). This is consistent with the site of earliest deposition of amyloid-β occurring intracellularly in the brains of people who have Down syndrome (Lemere *et al.*, 1996; Mori *et al.*, 2002; Cataldo *et al.*, 2004). A similar but non-significant increase in amyloid-β accumulation within trisomic;tgAPP granular cells of the dentate gyrus and in the cortex was also observed, but no change in full-length APP could be detected using similar methods (Supplementary Fig. 1). Consistent with this we saw a trend for an increase in the abundance of soluble aggregated amyloid-β, as measured by a multimeric amyloid-β ELISA, at 2 months of age in trisomic;tgAPP hippocampus as compared with tgAPP littermates (Fig. 1C).

We quantified plaque number at 6 and 16 months of age in the hippocampus in the trisomic × tgAPP model system. Trisomic;tgAPP progeny had significantly more extracellular plaques at both ages than tgAPP littermates (Fig. 1D–H). Similarly, the area of amyloid-β deposition was significantly greater in trisomic;tgAPP hippocampus and cortex compared with tgAPP controls at 16 months of age (Fig. 1I and Supplementary Fig. 2).

### Trisomy of chromosome 21 genes other than *APP* does not alter the ratio of amyloid-β isoforms that aggregate

**Figure 2:**
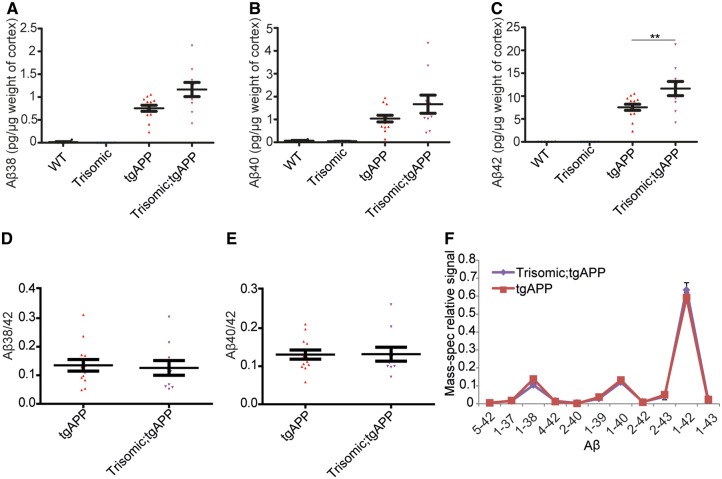
**Trisomy of chromosome 21 promotes the aggregation of amyloid-β.** (**A**–**F**) Cortical proteins from 16-month-old mice were fractionated and 5 M guanidine hydrochloride soluble amyloid-β quantified by (**A**–**E**) Meso Scale Discovery Assay or (**F**) mass spectrometry. (**A** and **B**) No effect of trisomy on amyloid-β_38_ [tgAPP-trisomy interaction *F*(1,28) = 0.385, *P = *0.540] or amyloid-β_40_ [tgAPP-trisomy interaction *F*(1,28) = 0.962, *P = *0.355] was observed, (**C**) but significantly more amyloid-β_42_ was detected in trisomic;tgAPP mice [tgAPP-trisomy interaction *F*(1,28) = 5.573, *P = *0.025] (Bonferroni pairwise comparisons trisomic;tgAPP with tgAPP *P = *0.005, wild-type *n = *6, trisomic = 6, tgAPP *n = *13, trisomic;tgAPP *n = *11). (**D** and **E**) No change in the amyloid-β_38_/_42_ ratio [trisomy *F*(1,19) = 0.072, *P = *0.792] or amyloid-β_40/42_ ratio [trisomy *F*(1,19) = 0.047, *P = *0.831] was observed. (**F**) The relative abundance of different forms of amyloid-β peptides was not altered by trisomy of chromosome 21 (tgAPP *n = *12, trisomic;tgAPP *n = *10). Data are represented as mean ± SEM, *P < 0.05, **P < 0.01, ***P < 0.001. Both male and female mice were used and sex was included as a factor in the ANOVA. Aβ = amyloid-β; WT = wild-type.

To determine which forms of amyloid-β peptide have increased deposition in the trisomic;tgAPP model, cortical proteins from 6- and 16-month-old animals were biochemically fractionated by homogenization in progressively more chemically disruptive solutions followed by ultracentrifugation (Tris buffer; then 1% Triton™ X-100; then 5 M guanidine hydrochloride) (Shankar *et al.*, 2009). The abundance of amyloid-β_42_, amyloid-β_40_ and amyloid-β_38_ in each fraction was then determined (Fig. 2A–C and Supplementary Fig. 3). At 16 months of age more amyloid-β_42_ was observed in the least soluble fraction (5 M guanidine hydrochloride) in trisomic;tgAPP compared with tgAPP controls, as measured using two independent assays (Fig. 2C and Supplementary Fig. 3F). No changes in the amyloid-β_40/42_ or amyloid-β_38/42_ ratios were observed in the aggregated (5 M guanidine hydrochloride soluble) fraction at either 6 or 16 months of age (Fig. 2D, E and Supplementary Fig. 3). Consistent with this observation, we found no difference in the relative abundance of amyloid-β species, as determined by mass spectrometry, between trisomic;tgAPP and tgAPP in the 5 M guanidine hydrochloride fraction isolated from 16-month-old cortex (Fig. 2F). Thus, trisomy of chromosome 21 sequences other than *APP*, are sufficient to greatly increase intracellular and extracellular amyloid-β deposition independently of changing the ratio of amyloid-β that aggregates.

### Trisomy of chromosome 21 genes other than *APP* exacerbates APP/amyloid-β associated cognitive deficits

**Figure 3:**
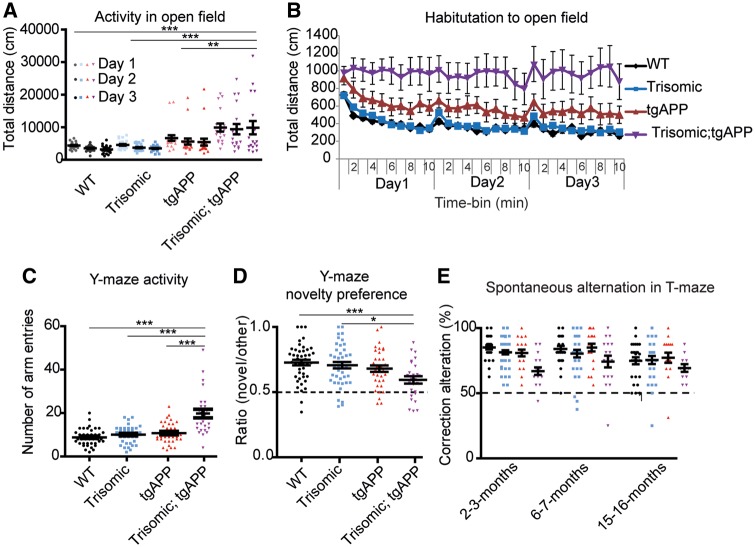
**Trisomy of chromosome 21 exacerbates APP/amyloid-β-associated cognitive deficits.** (**A** and **B**) Exposure to a novel open field was used as a test of activity and habituation (6 months, wild-type *n = *28, trisomic *n = *28, TgAPP *n = *20, trisomic;tgAPP *n = *18). (**A**) Overall activity: ANOVA of distance travelled revealed a main effect of tgAPP [*F*(1,78) = 26.250, *P < *0.001], trisomy [*F*(1,78) = 9.246, *P = *0.003], and a tgAPP-trisomy interaction [*F*(1,78) = 7.818, *P = *0.007] (Bonferroni pairwise comparisons trisomic;tgAPP with wild-type *P < *0.001, trisomic *P < *0.001, and tgAPP *P = *0.008). (**B**) The total distance moved declined with exposure time (1-min time bins) ANOVA: main effect of time bin [*F*(29,2262) = 12.399 *P < *0.001]; an interaction of time bin × trisomy [ANOVA *F*(29,2262) = 1.789 *P = *0.006], time bin × tgAPP [*F*(29,2262) = 1.560 *P = *0.029] and time bin × trisomy;tgAPP [*F*(29,2262) = 1.983, *P < *0.001] was observed by ANOVA. (**C** and **D**) A Y-maze spatial novelty preference task (1-min delay) was used as a test of activity and memory. (Cohort B 2–3 months and Cohort A 6–7 months, the effect of genotype was similar in both cohorts so data were combined for analysis, wild-type *n = *45, trisomic *n = *43, TgAPP *n = *36, trisomic;tgAPP *n = *26). (**C**) ANOVA of the number of arm entries (test phase), revealed a main effect of trisomy [*F*(1,89) = 50.360, *P < *0.001], tgAPP [*F*(1,89) = 47.001, *P < *0.001], and a tgAPP–trisomy interaction [*F*(1,89) = 31.720, *P < *0.001] (Bonferroni pairwise comparisons trisomic;tgAPP with wild-type *P < *0.001, trisomic *P < *0.001, and tgAPP *P < *0.001). (**D**) A preference ratio of 0.5 indicates chance performance (black dotted line). ANOVA of novelty preference revealed a main effect of trisomy [*F*(1,89) = 10.144 *P = *0.002], tgAPP [*F*(1,89) = 9.312 *P = *0.003] and a tgAPP–trisomy interaction [*F*(1,89) = 5.736, *P = *0.019] (Bonferroni pairwise comparisons trisomic;tgAPP with wild-type *P < *0.001 and trisomic *P = *0.010). Performance of tgAPP–trisomic mice was above chance (one-sample *t*-test t = 3.287 *P* < 0.001). (**E**) A discrete-trial, longitudinal spontaneous alternation task in a T-maze was used as a test of memory, 50% alternation represents chance performance (black dotted line) (2–3 months wild-type *n = *29, trisomic *n = *30, TgAPP *n = *21, trisomic;tgAPP *n = *17, 6–7 months wild-type *n = *28, trisomic *n = *29, TgAPP *n = *20, trisomic;tgAPP *n = *17, 15–16 months wld-type *n = *27, trisomic *n = *26, TgAPP *n = *27, trisomic;tgAPP *n = *11). ANOVA of alternation showed a main effect of trisomy [*F*(1,67) = 7.084 *P = *0.010], and an interaction of tgAPP–trisomy [*F*(1,67) = 4.706, *P* = 0.034] (Bonferroni pairwise comparison trisomic;tgAPP with wild-type *P = *0.032). Performance of tgAPP-trisomic mice was above chance (one-sample *t*-test, 2–3 months t = 5.884 *P* < 0.001, 6–7 months t = 5.378 *P* < 0.001, 15–16 months t = 6.495 *P* < 0.001). Data are represented as mean ± SEM, *P < 0.05, **P < 0.01, ***P < 0.001. Both male and female mice were used and sex was included as a variable in the ANOVA. Aβ = amyloid-β; WT = wild-type.

To determine if the chromosome 21 trisomy-associated increase in amyloid-β aggregation contributes to changes in APP/amyloid-β-associated cognitive deficits, a series of behavioural tests were undertaken on cohorts of wild-type, trisomic, tgAPP and trisomic;tgAPP littermates. These experiments were designed to avoid potential floor effects in test performance; so any interaction between the influence of trisomy and tgAPP status could be identified. Therefore tasks in which the trisomic mice had near-wild-type performance were used. This approach is also being developed to improve the assessment of cognitive decline in adults who have Down syndrome (Startin *et al.*, 2016). Trisomy of chromosome 21 significantly exacerbated APP/amyloid-β-associated hyperactivity at both 2–3 and 6–7 months of age, and trisomic;tgAPP animals specifically failed to habituate to an open field at 6–7 months of age (Fig. 3A–C and Supplementary Fig. 4A and B). Typical habituation, as measured by a decline in activity caused by increased familiarity with the new environment, was observed in wild-type, trisomic and tgAPP littermates (Fig. 3B). In this task we also observed that both trisomic and trisomic;tgAPP mice spent significantly more time in the centre of the open field than wild-type or tgAPP animals (Supplementary Fig. 4C). However, trisomic;tgAPP mice did not spend any additional time in the centre of the field compared with trisomic controls. Both groups of animals spent ∼20% of the time available in the centre of the field thus a ceiling effect is not biasing this task. These data suggest the failure to habituate in the trisomic;tgAPP is not driven by a modification of anxiety. We note that this task was not designed to measure anxiety; the open field and lighting conditions were designed to be non-anxious. However, these data indicate the Tc1-trisomic mice may have lower anxiety than wild-type controls, and further tests are warranted to explore this.

To understand the observed failure to habituate in the trisomic;tgAPP mice further, we undertook two tests of immediate memory. For the Y-maze task, two independent cohorts of mice were tested, one at 2–3 months of age and one at 6–7 months of age. The effect of genotype was similar in both cohorts so they were combined for analysis. For the T-maze task, one cohort of mice was tested longitudinally, at 2–3 months, 6–7 months and 15–16 months of age. We note that the performance of wild-type mice declined with age in this task, as has been previously reported (Lalonde, 2002). No deficit as measured by spatial novelty preference in the Y-maze (1-min trial interval) was observed in tgAPP or trisomic mice (Fig. 3D). However, trisomic;tgAPP mice had a poorer test performance than wild-type and trisomic animals in this task, such that a main effect of trisomy of chromosome 21 and of tgAPP was observed, and a significant interaction of these genetic factors was seen (Fig. 3D). Although some residual memory was retained, as trisomic;tgAPP had above chance performance in the task at the three ages studied. The poorer performance of trisomic;tgAPP was apparent despite these animals having a greater opportunity to experience extra-maze cues during the training phase than trisomic controls because of a greater time spent in the training (other) arm during the initial exposure to the maze (Supplementary Fig. 4B). An independent immediate memory task, a discrete-trial spontaneous alternation in the T-maze, was used to validate these observations. In this task, a main effect of chromosome 21 trisomy and an interaction of trisomy with tgAPP was observed by ANOVA, such that trisomic;tgAPP mice performed the task significantly worse than wild-type controls (Fig. 3E). Consistent with our observations in the Y-maze task, some residual memory was retained, as trisomic;tgAPP did have above chance performance at the three ages studied.

These data show that trisomy chromosome 21 exacerbates APP/amyloid-β associated cognitive changes, and that trisomic;tgAPP mice exhibit specific immediate memory deficits in two tasks compared to the matched controls. Additionally, trisomic;tgAPP mice were less likely to survive to 15 months of age than matched tgAPP control animals (Supplementary Fig. 4D). Thus, the chromosome 21 trisomy-associated increase in amyloid-β accumulation described here correlates with changes in multiple tests of cognition and is associated with an increased risk of mortality.

### Trisomy of chromosome 21 genes other than *APP* does not alter APP or β-CTF/α-CTF ratio in the brain

**Figure 4:**
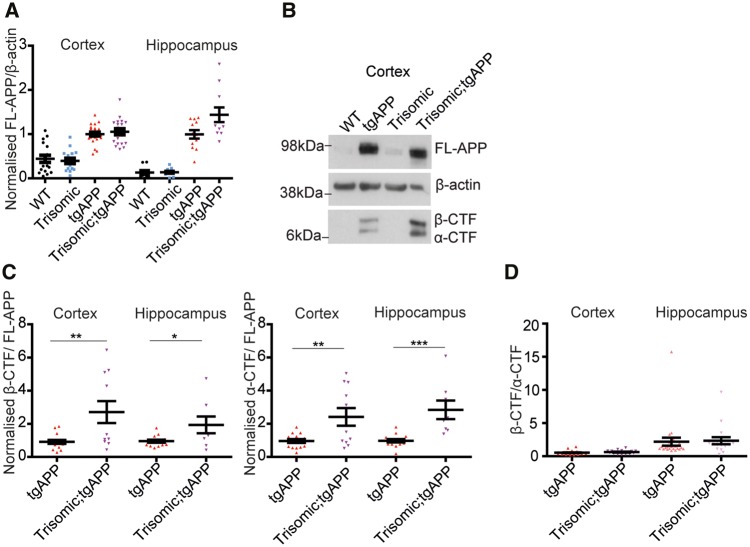
**Trisomy of chromosome 21 genes other than *APP* does not increase APP abundance nor alter β-CTF/α-CTF ratio.** (**A**, **B** and **D**) Full-length APP (FL-APP), APP β-CTF and APP α-CTF were measured in cortex (wild-type *n = *17, trisomic *n = *16, tgAPP *n = *24, trisomic;tgAPP *n = *19) and hippocampus (wild-type *n = *11, trisomic *n = *12, tgAPP *n = *24, trisomic;tgAPP *n = *17) at 3 months of age. (**A**) Full-length APP was higher in tgAPP and trisomic;tgAPP compared with wild-type or trisomic mice [cortex *F*(1,68) = 87.667, *P < *0.001, hippocampus *F*(1,56) = 94.301, *P < *0.001]. Trisomy did not alter full-length APP [trisomy–tgAPP interaction, cortex *F*(1,68) = 0.483, *P = *0.489, hippocampus *F*(1,56) = 2.457, *P = *0.123]. (**B** and **C**) In male mice, APP-CTF/full-length APP ratio was altered (cortex tgAPP *n = *17, trisomic;tgAPP *n = *11, hippocampus tgAPP *n = *14, trisomic;tgAPP *n = *8) β-CTF/full-length APP (*t*-test cortex *P = *0.005, hippocampus *P = *0.0217) and α-CTF/full-length APP (*t*-test cortex *P = *0.005 hippocampus *P < *0.001). (**D**) Trisomy did not alter the β-CTF/α-CTF ratio in the cortex [trisomy *F*(1,37) = 0.065, *P = *0.799] or hippocampus [trisomy *F*(1,37) = 1.082, *P = *0.305]. (**B**) Cropped western blot, four lanes of an eight-lane gel. Data are represented as mean ± SEM, *P < 0.05, **P < 0.01, ***P < 0.001. WT = wild-type.

To determine if a change in the abundance of APP in the brain of trisomic mice contributes to the increase in amyloid-β aggregation, we compared levels of full-length APP in cortex and hippocampus from progeny of the trisomic × tgAPP cross (3 months of age) (Fig. 4A and B). As expected, animals with tgAPP had higher levels of full-length APP protein, compared with the lower levels observed in both wild-type and trisomic controls. However, no significant increase in the abundance of full-length APP in the trisomic;tgAPP progeny compared with tgAPP controls was observed. Additionally, trisomic;tgAPP mice have comparable levels of human *APP* transcript compared with tgAPP in both the cortex and hippocampus (Supplementary Fig. 5). Mouse *App* mRNA levels in the trisomic, tgAPP and trisomic;tgAPP hippocampus were similar to wild-type mice (Supplementary Fig. 5), consistent with previous reports (Sheppard *et al.*, 2012; Gribble *et al.*, 2013). Thus, trisomy does not cause exacerbated amyloid-β deposition in our model system by increasing the abundance of full-length APP protein *in vivo.*

Alterations in the processing of APP by α- then γ-secretase or β- then γ-secretase, can modulate amyloid-β deposition (Haass *et al.*, 2012). Cleavage of full-length APP by β-secretase generates soluble β-APP and a membrane bound β-C-terminal fragment (β-CTF), which can then be cleaved by γ-secretases, forming amyloid-β. Whereas cleavage of APP by an α-secretase, produces soluble α-APP and α-C-terminal-fragment (α-CTF), in a process that prevents β-cleavage and hence the formation of amyloid-β. We found that chromosome 21 trisomy causes a significant increase in both α- and β-CTFs relative to full-length APP, in both the hippocampus and cortex at 3 months of age in male mice (Fig. 4B and C). However, no significant change in α- or β-CTF/full-length APP ratios occurs in female mice (Supplementary Fig. 6). Moreover, trisomy of chromosome 21 did not alter the β-CTF/α-CTF ratio in either sex in the cortex or hippocampus (Fig. 4D). This indicates that although trisomy of chromosome 21 increases APP-CTF abundance in males, it does not alter the relative balance of the amylogenic versus non-amylogenic APP processing pathway in the brain. Additionally, trisomy-associated increases in amyloid-β accumulation are observed in both males and females, suggesting that the specific effect of trisomy in male mice on APP-CTF abundance is unlikely to be the cause of enhanced amyloid-β deposition observed in our mouse model system.

### Trisomy of chromosome 21 genes other than *APP* does not elevate APP-CTF production

**Figure 5:**
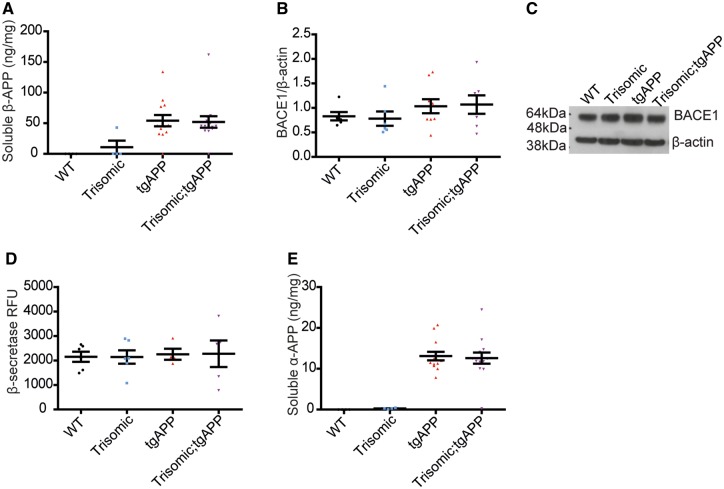
**Trisomy of chromosome 21 genes other than *APP* does not alter α-/β-secretase activity.** Trisomy did not alter (**A**) soluble β-APP abundance [trisomy *F*(1,24) = 0.790, *P = *0.383, trisomy-tgAPP interaction *F*(1,24) = 0.773, *P = *0.388, wild-type *n = *4, trisomic *n = *4, TgAPP *n = *13, trisomic;tgAPP *n = *14], (**B** and **C**) cortical BACE1 abundance [trisomy *F*(1,24) = 0.002, *P = *0.963; trisomy–tgAPP interaction *F*(1,24) = 0.071, *P = *0.792, wild-type *n = *6, trisomic *n = *6, TgAPP *n = *9, trisomic;tgAPP *n = *7], (**D**) BACE1 β-secretase activity [trisomy *F*(1,13) = 0.006, *P = *0.941; trisomy–tgAPP interaction *F*(1,13) = 0.001 *P = *0.971, wild-type *n = *6, trisomic *n = *6, TgAPP *n = *4, trisomic;tgAPP *n = *5], or (**E**) soluble α-APP abundance [trisomy *F*(1,27) = 0.041, *P = *0.841; trisomy–tgAPP interaction *F*(1,27) = 0.002, *P = *0.969, wild-type *n = *4, trisomic *n = *4, TgAPP *n = *13, trisomic;tgAPP *n = *14]. (**C**) Cropped western blot, four lanes of an eight-lane gel. Data are represented as mean ± SEM. Both sexes were analysed and sex was included as a factor in the ANOVA. Aβ = amyloid-β.

To determine if the elevation in APP-CTFs in males is caused by an increased rate of CTF-production; we analysed the abundance of soluble β-APP, the other APP fragment produced by β-secretase activity. Trisomy of chromosome 21 did not alter the abundance of soluble β-APP in the cortex of the Tc1 mouse (Fig. 5A), indicating that the production of β-CTF is not upregulated *in vivo* by trisomy of chromosome 21. Consistent with this, BACE1 level and BACE1-β-secretase activity (as measured by a BACE1 capture enzymatic assay) were not altered by trisomy at 3 months of age, in the cortex (Fig. 5B–D). Similarly, trisomy of Hsa21 did not affect levels of soluble α-APP in the cortex at 3 months of age (Fig. 5E). Therefore, the elevated β-CTF and α-CTF/full-length APP ratios observed in male trisomic;tgAPP compared with tgAPP male controls are likely the result of impaired turn-over of these fragments.

### Trisomy of chromosome 21 does not alter the extracellular clearance of amyloid-β

**Figure 6:**
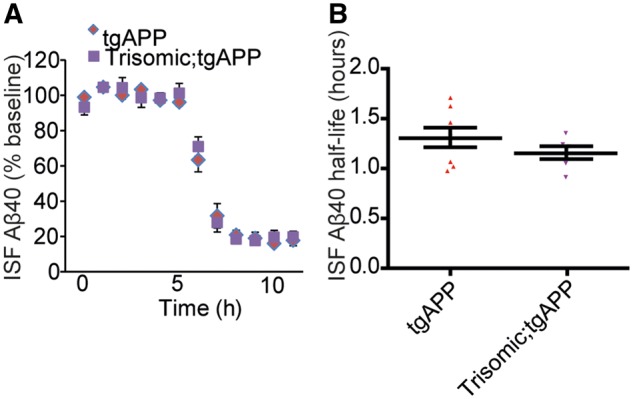
**Trisomy of chromosome 21 does not alter the half-life of extracellular amyloid-β.** (**A** and **B**) The *in vivo* half-life of amyloid-β_40_, measured by microdialysis of hippocampal interstitial fluid (ISF), was not altered by trisomy of chromosome 21. Compound E injected at 6 h to halt further amyloid-β generation (*t*-test *P = *0.258, tgAPP *n = *8, trisomic;tgAPP *n = *6 females only). Data are represented as mean ± SEM. Aβ = amyloid-β.

The increase in abundance of β-CTF and α-CTF in trisomic male mice indicates that trisomy of chromosome 21 may alter the clearance of APP derivatives in some circumstances. To determine if trisomy causes increased amyloid-β aggregation by impairing clearance of the peptide *in vivo*, we measured the half-life of extracellular amyloid-β_40_ in the hippocampus at 3 months of age by microdialysis of interstitial fluid. To follow amyloid-β clearance kinetics *in vivo*, a potent inhibitor of γ-secretase (compound E) was injected to halt amyloid-β generation. Chromosome 21 trisomy did not significantly alter amyloid-β_40_ half-life (Fig. 6A and B), nor did it alter the expression of the key amyloid-β extracellular clearance enzymes, insulin degrading enzyme and neprilysin in the brain (Supplementary Fig. 7). Thus changes in the extracellular clearance of amyloid-β do not contribute to trisomy-associated increases in deposition.

### Trisomy of chromosome 21 genes other than *APP* causes a shift in the soluble amyloid-β_40/42_ ratio

**Figure 7:**
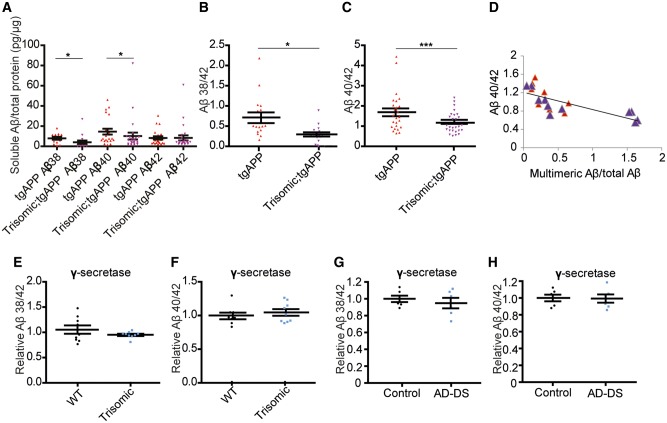
**Trisomy of chromosome 21 modulates amyloid-β ratios *in vivo* independently of modulation of γ-secretase activity.** (**A**–**C**) Tris-soluble amyloid-β_38_, amyloid-β_40_ and amyloid-β_42_ were measured by Meso Scale Discovery 6E10 Aβ Triplex assay in 2-month-old hippocampus (wild-type *n = *24, trisomic *n = *22, TgAPP *n = *25 and trisomic;tgAPP *n = *27). (**A**) Trisomy decreased amyloid-β_38_ [trisomy *F*(1,4) = 15.403, *P = *0.017] and amyloid-β_40_ [trisomy *F*(1,11) = 6.359, *P = *0.028], but amyloid-β_42_ was not changed [trisomy *F*(1,11) = 2.978, *P = *0.112], (**B**) resulting in an alteration in the amyloid-β_38/42_ ratio [trisomy *F*(1,4) = 14.553, *P = *0.019] and (**C**) the amyloid-β_40/42_ ratio [trisomy *F*(1,10) = 95.694, *P < *0.001]. (**D**) Hippocampal amyloid-β_40/42_ ratio negatively correlates with the relative abundance of aggregated amyloid-β (multimeric 82E1-82E1 amyloid-β ELISA) (linear correlation, R^2 ^= 0.5485, *P = *0.0003, tgAPP *n = *8, trisomic;tgAPP *n = *11). Trisomy did not alter the carboxypeptidase activity of the γ-secretase complex, as measured by (**E** and **G**) amyloid-β_38_/_42_ and (**F** and **H**) amyloid-β_40/42_ ratios produced *in vitro* by the complex isolated from cortex from (**E** and **F**) the Tc1 trisomic mouse [trisomy amyloid-β_38/42 _*F*(1,11) = 4.88, *P = *0.499; amyloid-β_42/40 _*F*(1,11) = 0.799, *P = *0.395, wild-type = 9, trisomic = 9] and (**G** and **H**) people with Down syndrome and Alzheimer’s disease (AD-DS) (*n = *6) compared with age- and sex-matched individuals who did not have Down syndrome or dementia (control *n = *6) [trisomy amyloid-β_38/42 _*F*(1,5) = 0.102, *P = *0.763; amyloid-β_42/40 _*F*(1,5) = 0.187, *P = *0.684]. (**A**–**F**) Data are represented as mean ± SEM, (**G** and **H**) individual cases plotted, horizontal line indicates mean ± SEM, *P < 0.05, **P < 0.01, ***P < 0.001. Both male and females were studied and sex was included as a variable in the ANOVA. Aβ = amyloid-β; WT = wild-type.

The aggregation rate of amyloid-β *in vitro* is influenced by the relative abundance of more aggregate prone amyloid-β species, such as amyloid-β_42_, compared with forms of the peptide, which are less able to seed aggregation such as amyloid-β_40_ (Vandersteen *et al.*, 2012). To determine if chromosome 21 trisomy associated increases in amyloid-β deposition are the result of changes in the ratio of amyloid-β species *in vivo*, we investigated the relative abundance of soluble amyloid-β prior to accumulation of detectable amyloid-β deposits in the hippocampus of 2–3-month-old progeny from the trisomic × tgAPP cross. Levels of Tris-soluble total hippocampal amyloid-β_42_ were not affected by trisomy, but a significant decrease in both tris-soluble amyloid-β_38_ and amyloid-β_40_ was observed, leading to a significant decrease in the soluble amyloid-β_38/42_ and amyloid-β_40/42_ ratios (Fig. 7A–C). Moreover the soluble amyloid-β_40/42_ ratio significantly correlates with amyloid-β aggregation in young hippocampus, as measured using a multimeric amyloid-β ELISA (Fig. 7D), indicating that the effect of trisomy on soluble amyloid-β ratios may underlie the increased accumulation of the peptide observed within the brain.

### Trisomy of chromosome 21 genes other than *APP* does not alter intrinsic γ-secretase carboxypeptidase activity

Impairment of γ-secretase carboxypeptidase activity, such as is caused by some familial Alzheimer’s disease mutations in *APP* or *PSEN1* or *PSEN2*, can result in similar changes to the amyloid-β isoform ratios (Chavez-Gutierrez *et al.*, 2012). We isolated a membrane fraction containing γ-secretase from the cortex of our mouse model and used this for an *in vitro* enzymatic activity assay using a recombinant APP-CTF substrate. No change in γ-secretase carboxypeptidase activity was detected in the trisomic mouse model (Fig. 7E and F). Similarly using the same method, no difference in enzymatic activity was observed in people who have Down syndrome–Alzheimer’s disease compared with aged-matched healthy controls (Fig. 7G, H, and Supplementary Table 1). Trisomy of chromosome 21 therefore alters the amyloid-β ratio *in vivo* independently of a direct effect on γ-secretase activity.

## Discussion

The extra copy of *APP*, encoded on chromosome 21, has a central role in Down syndrome-Alzheimer’s disease pathogenesis. This includes increasing the level of APP protein and its derivatives, such as amyloid-β, and causing specific alteration to endolysosomal biology (Lemere *et al.*, 1996; Salehi *et al.*, 2006; Cheon *et al.*, 2008; Jiang *et al.*, 2010). Here we show that in addition, trisomy of other chromosome 21 sequences is sufficient to promote the aggregation and deposition of amyloid-β within the brain and worsen associated-cognitive deficits. Our data suggest that the increase in amyloid-β aggregation caused by trisomy of chromosome 21 may be mediated by an alteration in the ratio of soluble amyloid-β isoforms that occurs independently of alterations in the activity of α-, β- or γ- secretases, or a change in the rate of extracellular clearance of amyloid-β.

Our research uses animal models created to study Down syndrome and amyloid-β accumulation, to investigate the effect of triplication of chromosome 21 genes on amyloid-β generation and deposition within the brain. As with all work using animal models, it is essential to recognize that each model gives us a limited view of a complex human disease. Down syndrome mouse models in which *App* is triplicated [e.g. Ts65Dn or *Dp(10)1Yey;Dp(16)1Yey;Dp(17)1Yey* triple trisomic mice] do not form amyloid-β plaques even in elderly mice (26 months) (Reeves *et al.*, 1995; Yu *et al.*, 2010). Thus, to understand the development of amyloid pathology in the context of chromosome 21 trisomy we work with a widely-used transgenic *APP* mouse model. This model overexpresses human APP with Alzheimer’s disease associated point mutations, which promote the formation of aggregation prone amyloid-β_42_, and develops robust amyloid pathology. Therefore in this model system, the relative abundance of amyloid-β_42_ is higher than would be typically observed in individuals who have Down syndrome.

We crossed the tgAPP mouse with the Tc1 mouse model of Down syndrome that contains a freely-segregating copy of human chromosome 21. This model system allows us to address whether the additional copy of the chromosome 21 genes carried in the Tc1 mouse are sufficient to alter amyloid-β generation, deposition and associated cognitive changes. However, we note that as with all models of Down syndrome (including the triple trisomics) (Yu *et al.*, 2010), the Tc1 mouse does not have the full complement of chromosome 21 sequences (Gribble *et al.*, 2013). Indeed, here we have taken advantage of the absence of an additional copy of *APP* in this model to investigate *APP* triplication-independent effects. Within the Tc1 mouse model the transchromosome is lost during development from some cells; we previously determined that ∼66% of brain nuclei retain the chromosome (O’Doherty *et al.*, 2005). Chromosome 21 mosaicism, in individuals who have Down syndrome, is associated with higher IQ, earlier acquisition of developmental milestones, less severe cardiac defects and reduced mortality compared to matched cases of non-mosaic chromosome 21 trisomy (Papavassiliou *et al.*, 2009; Zhu *et al.*, 2013). Similarly, in the Tc1 mouse, mosaicism may result in a reduction in phenotypic severity compared with that in non-mosaic Down syndrome model systems. Further studies in alternative mouse models of Down syndrome that are not mosaic, in particular mice with segmental duplications of the mouse genome orthologous to regions of human chromosome 21, may help investigate this (Brault *et al.*, 2006; Yu *et al.*, 2010; Lana-Elola *et al.*, 2011, 2016).

Our results demonstrate that a number of cognitive changes are observed in trisomic;tgAPP mice compared to wild-type controls. In particular, these mice display a profound hyperactivity, a failure to habituate to a novel open-field, and poorer immediate memory than wild-type controls. These changes may relate to the increase in amyloid-β aggregation observed in these mice or how trisomic neurons respond to aggregating amyloid-β. Further studies, in which a trisomic model and wild-type control are exposed to equal quantity of amyloid-β are required to understand the mechanisms that cause the changes in cognition observed in our study.

Our findings suggest a number of new avenues of inquiry that warrant further investigation. Our work indicates that people who have Down syndrome may have exacerbated amyloid accumulation compared with individuals who have early-onset Alzheimer’s disease caused by duplication of *APP.* Comparative pathological studies of the two causes of early-onset disease are required to investigate this hypothesis. Notably, comparative studies of Alzheimer’s disease in people who have Down syndrome and sporadic Alzheimer’s disease have suggested that higher levels of aggregated amyloid-β accumulate in people who have Down syndrome (Hyman *et al.*, 1995; Egensperger *et al.*, 1999; Wilcock *et al.*, 2015), consistent with the finding in our mouse model system presented here. If amyloid deposition is found to be higher in people who have Down syndrome, understanding which gene on chromosome 21 other than *APP* contributes to this will provide novel insights into disease development and may provide a new target for drug therapy for individuals who have Down syndrome, who are at extraordinarily high risk of developing dementia.

Alzheimer’s disease in people who have Down syndrome first presents with memory impairment, behavioural changes, myoclonus and seizures; with a low incidence of cerebral haemorrhage and stroke. A recent review has noted that this pattern of clinical presentation is more similar to that seen in familial cases of Alzheimer’s disease caused by mutations in *APP* that decrease amyloid-β_40/42_ ratio, than to cases of diseases caused by duplication of *APP* (Zis and Strydom, 2018). The authors hypothesized that a shift to a lower amyloid-β_40/42_ ratio in people who have Down syndrome might explain this apparent clinical disparity. Our animal work supports this hypothesis and suggests further studies of the ratio of amyloid-β_40/42_ in people who have Down syndrome, particularly at the earliest stages of disease is warranted.

We first observe a significant increase in amyloid-β accumulation within CA3 pyramidal hippocampal neurons of our mouse model (Fig. 1B). A previous study using the Tc1 mouse showed that the CA3 has a particular vulnerability to trisomy of chromosome 21, which includes a specific reduction in synapse number, alterations to synapse architecture and related electrophysiological and behavioural deficits (Witton *et al.*, 2015). The sensitivity of the CA3 to trisomy of chromosome 21 is also observed in an alternative Down syndrome mouse model (Popov *et al.*, 2011). The specific effect of trisomy of chromosome 21 on the synapses of CA3 cells may contribute to their vulnerability to intracellular amyloid-β accumulation, as the synapse is proposed to be a key site of amyloid-β formation (Das *et al.*, 2013). Further work is required to determine if CA3 pyramidal cells in people who have Down syndrome also have a similar tendency to develop intracellular amyloid-β accumulation and the molecular changes that are responsible for this.

Additionally, this work also demonstrates that factors other than the intrinsic carboxypeptidase activity of the γ-secretase complex or the APP protein sequence can substantially alter the ratio of amyloid-β isoforms generated *in vivo.* Moreover, we show that an extra dose of a gene or genes encoded on chromosome 21 is sufficient to modulate this process. Identification of these gene/genes will provide novel insights into the pathways that can alter amyloid-β generation and how these processes could be modulated to prevent disease.

A number of proteins encoded on chromosome 21 have been suggested to influence amyloid-β biology and are trisomic in the Tc1 mouse model. These include *SUMO3*, which is conjugated to proteins to regulate their function and may influence APP processing (Li *et al.*, 2003; Dorval *et al.*, 2007). The kinase *DYRK1A* can phosphorylate APP and alter the protein’s stability and the formation of amyloid-β (Ryoo *et al.*, 2008; Garcia-Cerro *et al.*, 2017). Recently, a novel inhibitor of DYRK1A has been shown to reduce amyloid-β plaque load in an Alzheimer’s disease mouse model (Branca *et al.*, 2017). *BACE2*, a homologue of *BACE1* located on chromosome 21 may impair the formation of amyloid-β by cleaving APP within the amyloid-β region (Sun *et al.*, 2006; Mok *et al.*, 2014). An endogenous inhibitor of lysosomal cathepsins, *CSTB*, which reduces amyloid-β accumulation when knocked-out, is also found on chromosome 21 (Yang *et al.*, 2011). Reduced activity of the enzymes inhibited by CSTB have been linked to altered clearance of amyloid-β and processing of APP; including a shift in the amyloid-β_40/42_ ratio (Mueller-Steiner *et al.*, 2006; Hook *et al.*, 2009; Wang *et al.*, 2015).

Moreover, recent studies have highlighted that alterations in γ-secretase trafficking or modulation of the endo-lysosome system can profoundly affect amyloid-β generation (Xiao *et al.*, 2015; Sannerud *et al.*, 2016); other as yet unidentified gene/genes on chromosome 21 may affect these processes and mediate the effect of trisomy on amyloid-β ratios, aggregation and deposition observed here. Identification of the causal gene(s) on chromosome 21 will provide further novel insights into the new Down syndrome–Alzheimer’s disease mechanism described in this study.

## Supplementary Material

Supplementary Data

## Abbreviation

CTF: C-terminal fragment

## Appendix 1

The LonDownS Consortium principal investigators are:

Andre Strydom, Department of Forensic and Neurodevelopmental Sciences, Institute of Psychiatry, Psychology and Neuroscience, King’s College London, London, UK and Division of Psychiatry, University College London, London, UK; Elizabeth Fisher, Department of Neurodegenerative Disease, UCL Institute of Neurology, London, UK; Dean Nizetic, Blizard Institute, Barts and the London School of Medicine, Queen Mary, University of London, London, UK, and Lee Kong Chian School of Medicine, Nanyang Technological University, Singapore, Singapore; John Hardy, Reta Lila Weston Institute, Institute of Neurology, University College London, London, UK, and UK Dementia Research Institute at UCL, London, UK; Victor Tybulewicz, Francis Crick Institute, London, UK and Department of Medicine, Imperial College, London, UK; and Annette Karmiloff-Smith (Birkbeck University of London, deceased).
