# Corrigendum

**DOI:** 10.1093/brain/awz061

**Published:** 2019-03-15

Frances K. Wiseman, Laura J. Pulford, Chris Barkus, Fan Liao, Erik Portelius, Robin Webb, Lucia Chávez-Gutiérrez, Karen Cleverley, Sue Noy, Olivia Sheppard, Toby Collins, Caroline Powell, Claire J. Sarell, Matthew Rickman, Xun Choong, Justin L. Tosh, Carlos Siganporia, Heather T. Whittaker, Floy Stewart, Maria Szaruga, London Down syndrome consortium, Michael P. Murphy, Kaj Blennow, Bart de Strooper, Henrik Zetterberg, David Bannerman, David M. Holtzman, Victor L. J. Tybulewicz, Elizabeth M. C. Fisher. Trisomy of human chromosome 21 enhances amyloid-β deposition independently of an extra copy of *APP*. Brain Volu 2018; 141: 2457–2474, https://doi.org/10.1093/brain/awy159.

The authors would like to apologise for an error in Fig. 4B in the manuscript as originally published. The figure appears correctly below. 

**Figure 4:**
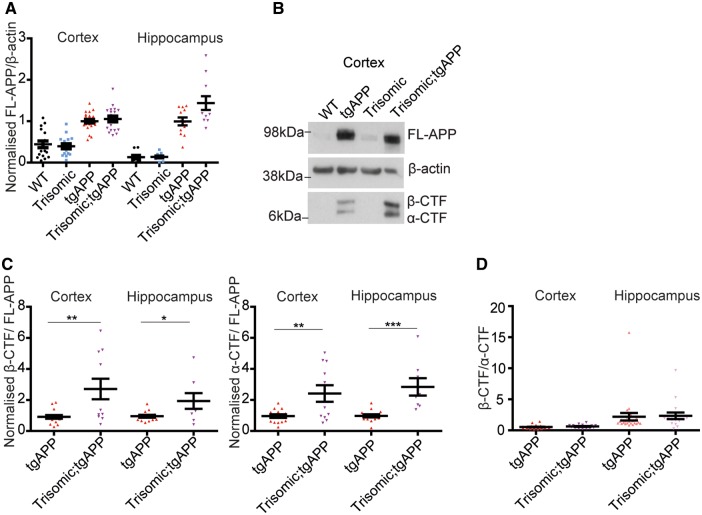
**Trisomy of chromosome 21 genes other than *APP* does not increase APP abundance nor alter β-CTF/α-CTF ratio.** (**A**, **B** and **D**) Full-length APP (FL-APP), APP β-CTF and APP α-CTF were measured in cortex (wild-type *n = *17, trisomic *n = *16, tgAPP *n = *24, trisomic;tgAPP *n = *19) and hippocampus (wild-type *n = *11, trisomic *n = *12, tgAPP *n = *24, trisomic;tgAPP *n = *17) at 3 months of age. (**A**) Full-length APP was higher in tgAPP and trisomic;tgAPP compared with wild-type or trisomic mice [cortex *F*(1,68) = 87.667, *P < *0.001, hippocampus *F*(1,56) = 94.301, *P < *0.001]. Trisomy did not alter full-length APP [trisomy–tgAPP interaction, cortex *F*(1,68) = 0.483, *P = *0.489, hippocampus *F*(1,56) = 2.457, *P = *0.123]. (**B** and **C**) In male mice, APP-CTF/full-length APP ratio was altered (cortex tgAPP *n = *17, trisomic;tgAPP *n = *11, hippocampus tgAPP *n = *14, trisomic;tgAPP *n = *8) β-CTF/full-length APP (*t*-test cortex *P = *0.005, hippocampus *P = *0.0217) and α-CTF/full-length APP (*t*-test cortex *P = *0.005 hippocampus *P < *0.001). (**D**) Trisomy did not alter the β-CTF/α-CTF ratio in the cortex [trisomy *F*(1,37) = 0.065, *P = *0.799] or hippocampus [trisomy *F*(1,37) = 1.082, *P = *0.305]. (**B**) Cropped western blot, four lanes of an eight-lane gel. Data are represented as mean ± SEM, *P < 0.05, **P < 0.01, ***P < 0.001. WT = wild-type.

The manuscript has been corrected online.

