# Supplementary material for: Trisomy of human chromosome 21 enhances amyloid-β deposition independently of an extra copy of APP
Source: Brain. 2018 Jun 26;141(8):2457–74. doi: 10.1093/brain/awy159 (PMC6061702; doi:10.1093/brain/awy159)
Supplement: Supplementary Data [file awy159_supplementary_data.pdf]

Supplementary Figure 1

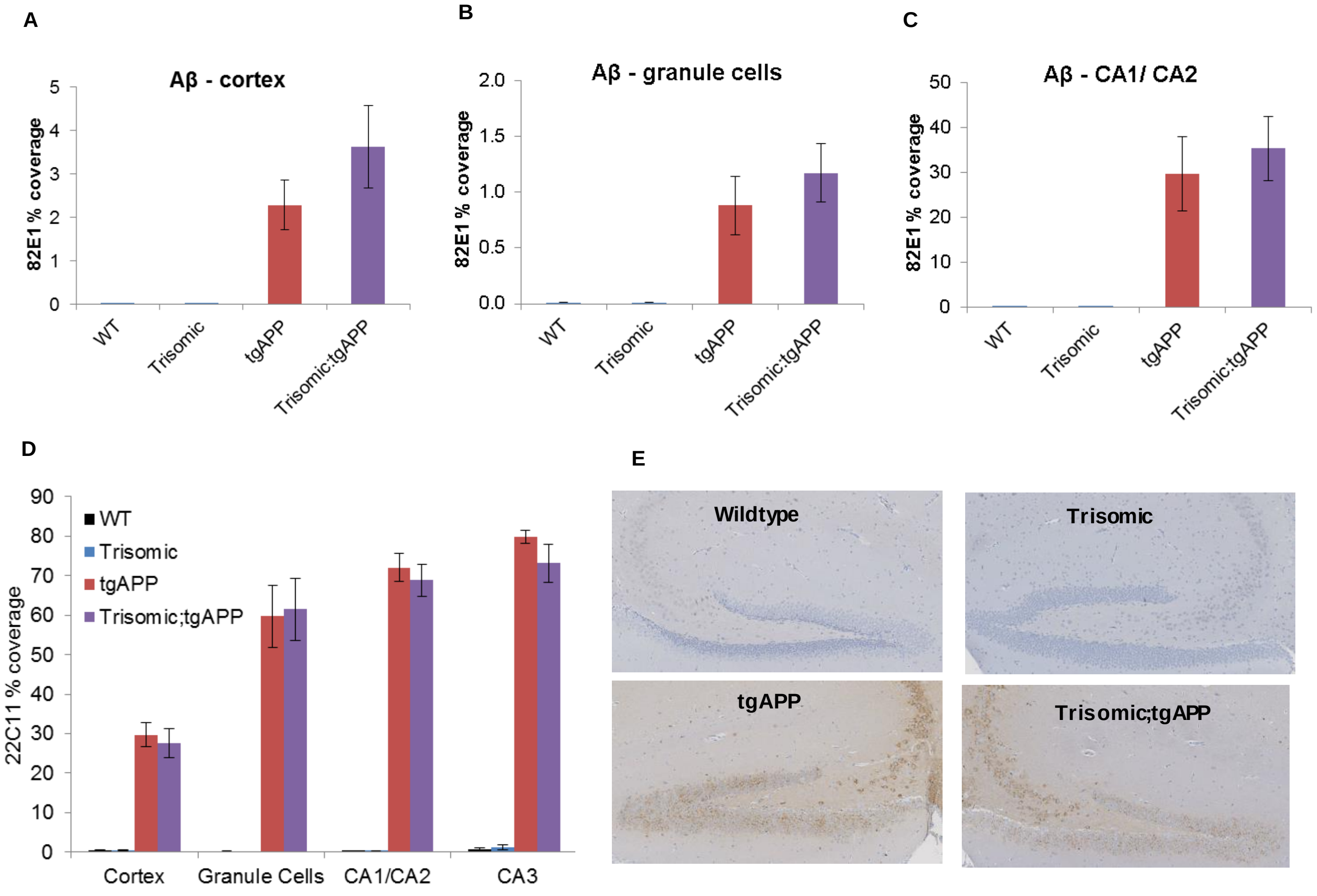

Supplementary Fig. 1: Aβ and FL-APP staining in the hippocampus and cortex

(**A-E**) Aβ and APP staining in the brain of wildtype (WT), Trisomic, tgAPP and Trisomic;tgAPP mice was quantified by immunohistochemistry, using (**A,B,C**) anti-Aβ antibody (82E1) and (**D,E**) anti-APP antibody (22C11). Mice were between 4.5-6.5-months of age (mean age for groups: WT = 146 days, Trisomic = 162 days, tgAPP = 159 days, Trisomic;tgAPP = 144 days). Trisomy of chromosome 21 did not significantly alter the area covered by Aβ in (**A**) cortex (trisomy-tgAPP interaction $F(1,31) = 1.267$, $p = 0.269$), (**B**) the granule cells of the dentate gyrus (trisomy-tgAPP interaction $F(1,31) = 2.781$, $p = 0.105$), (**C**) or in CA1/CA2 pyramidal cells (trisomy-tgAPP interaction $F(1,31) = 0.730$, $p = 0.399$). (**D,E**) Trisomy of chromosome 21 did not significantly alter the area covered by APP staining in cortex (trisomy-tgAPP interaction $F(1,31) = 0.053$, $p = 0.819$), the granule cells of the dentate gyrus (trisomy-tgAPP interaction $F(1,31) = 0.096$, $p = 0.756$), CA1/CA2 pyramidal cells (trisomy-tgAPP interaction $F(1,31) = 0.475$, $p = 0.496$) or CA3 pyramidal cells (trisomy-tgAPP interaction $F(1,31) = 2.931$, $p = 0.097$). Data show mean ± SEM. Both sexes were studied and sex was included as a variable in the ANOVA.

Supplementary Figure 2

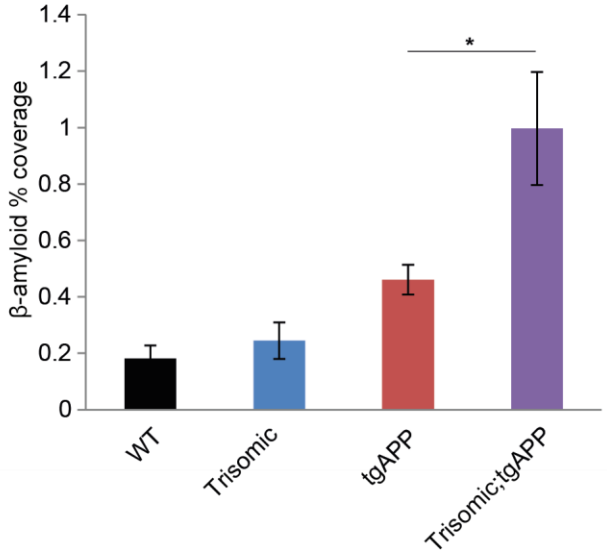

Supplementary Fig. 2: Aβ deposition in the cortex
Aβ deposition in the cortex of wildtype (WT), Trisomic, tgAPP and Trisomic;tgAPP mice was quantified using anti-β-amyloid antibody (6F/3D) at 16-months of age. Trisomy of chromosome 21 increases the area covered (ANOVA tgAPP-trisomy interaction $F(1,37) = 5.803$, $p = 0.021$). Data show mean ± SEM, $p < 0.05$ = *. Both sexes were studied and sex was included as a variable in the ANOVA.

Supplementary Figure 3

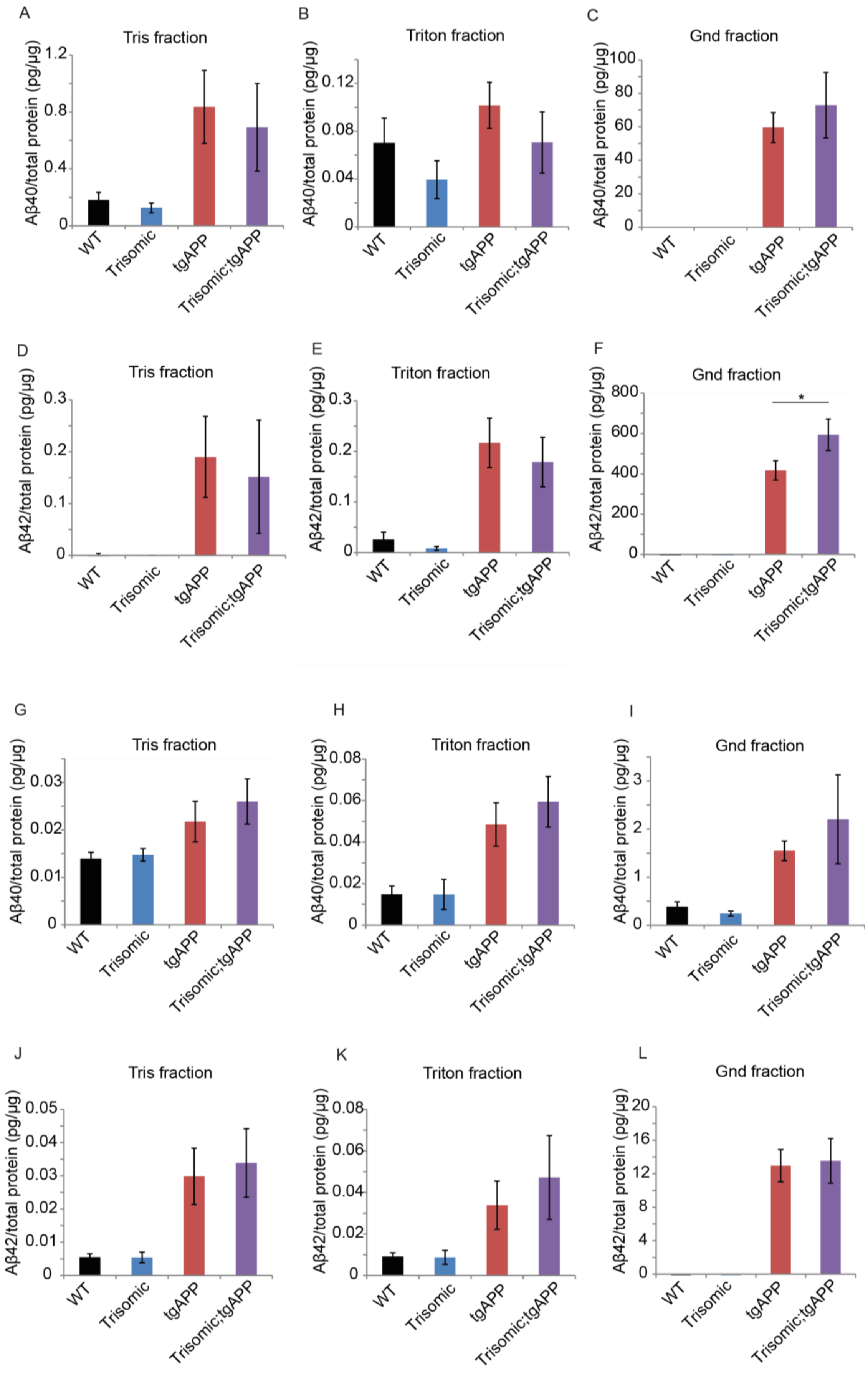

Supplementary Fig. 3: Human Aβ40 and Aβ42 in fractionated cortex

Total cortical proteins, from (**A-F**) 16-months or (**G-L**) 6-months of age wildtype (WT), Trisomic, tgAPP and Trisomic;tgAPP mice, were fractionated into (**A,D,G,J**) tris soluble, (**B,E,H,K**) 1 % triton soluble, and (**C,F,I,L**) 5 M guanidine hydrochloride (Gnd) soluble fractions. The amount of human (**A,B,C,G,H,I**) Aβ40 and (**D,E,F,J,K,L**) Aβ42 was quantified by ELISA. (**F**) Trisomy of chromosome 21 significantly increased the amount of 5 M guanidine hydrochloride soluble Aβ42 (ANOVA interaction of tgAPP-trisomy-age $F(1,40) = 5.037$ $p= 0.030$) at 16-months of age. No other statistically significant effects of genotype were observed. Data show mean ± SEM, $p < 0.05$ = *. Both sexes were studied and sex was included as a variable in the ANOVA.

Supplementary Figure 4

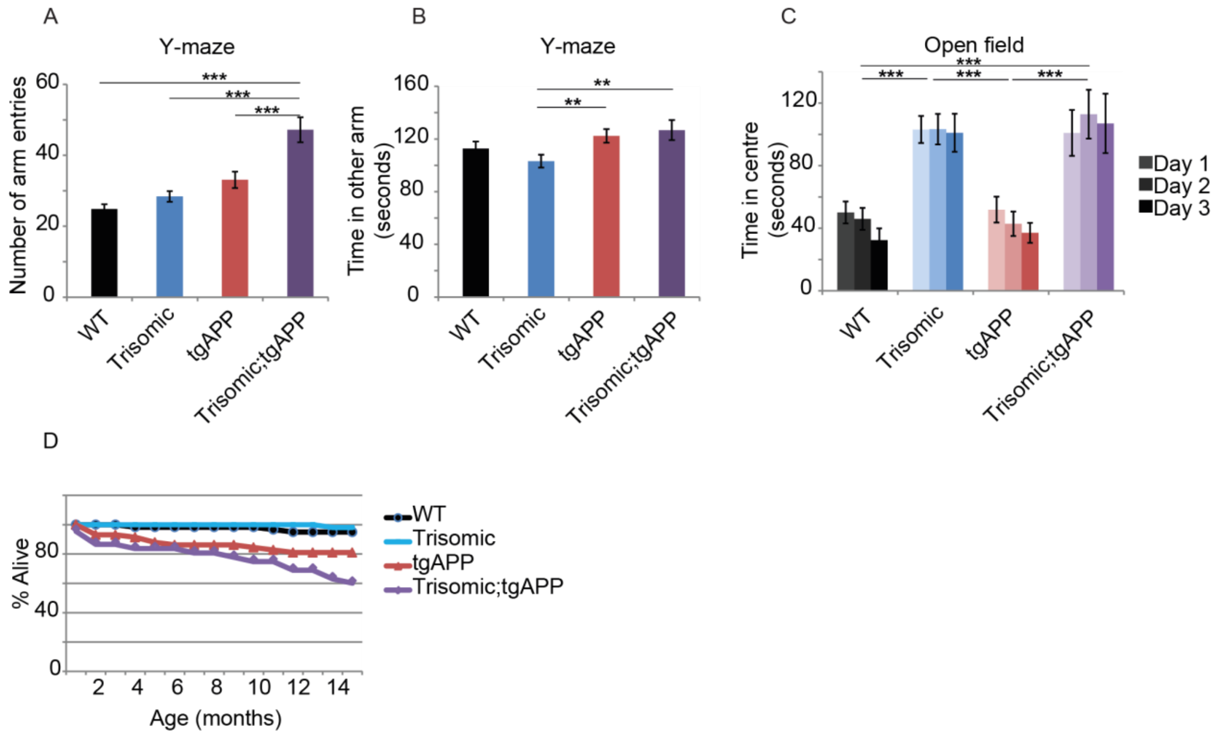

Supplementary Fig. 4: Y-maze exposure phase, open field control data and survival time
(**A**) The number of arm entries made during this first exposure to the Y-maze was increased in the Trisomic-tgAPP mice, ANOVA revealed a main effect of trisomy ($F(1,89) = 17.604$, $p<0.001$), of tgAPP ($F(1,89) = 28.519$, $p < 0.001$) and also an interaction of trisomy-tgAPP ($F(1,89) = 4.705$, $p = 0.033$)(Bonferroni pairwise comparisons; Trisomic;tgAPP with WT $p < 0.001$, Trisomic $p < 0.001$ and tgAPP $p < 0.001$, and tgAPP with WT $p = 0.011$). Cohort B 2-3-months of age and Cohort A 6-7-months of age, the effect of genotype was similar in both cohorts so data was combined for analysis, WT n = 45, Trisomic n = 43, TgAPP n = 36, Trisomic;tgAPP n = 26. (**B**) The total time spent in the other arm (non-start arm) during the Y-maze exposure phase showed a main effect of tgAPP ($F(1,89) = 9.570$, $p = 0.003$) and an interaction of tgAPP-trisomy ($F(1,89) = 6.972$, $p = 0.010$). (Bonferroni pairwise comparisons Trisomic with tgAPP $p = 0.008$ and Trisomic;tgAPP $p = 0.002$). (**C**) Time spent in the centre of the open field was affected by Trisomy at 6-months of age (ANOVA main effect $F(1,78) = 39.044$, $p < 0.001$, Bonferroni pairwise comparisons Trisomic with WT $p < 0.001$ and tgAPP $p < 0.001$, Trisomic;tgAPP with WT $p < 0.001$ and tgAPP $p < 0.001$). No interaction of Trisomy and tgAPP was found ($F(1,78) = 0.074$, $p = 0.786$). Day of exposure to the open field did not significantly alter time in the centre ($F(1,78) = 3.106$, $p = 0.082$) (**D**) Survival to 15 months of age was reduced in Trisomic;tgAPP compared with tgAPP mice (Chi-squared = 3.88, $p = 0.048$, Cohort A and C WT n = 60, Trisomic n = 53, TgAPP n = 58, Trisomic;tgAPP n = 34). Data are represented as mean ± SEM, * $p < 0.05$, ** $p < 0.01$, *** $p < 0.001$. Both sexes were studied and sex was included as a variable in the ANOVA.

Supplementary Figure 5

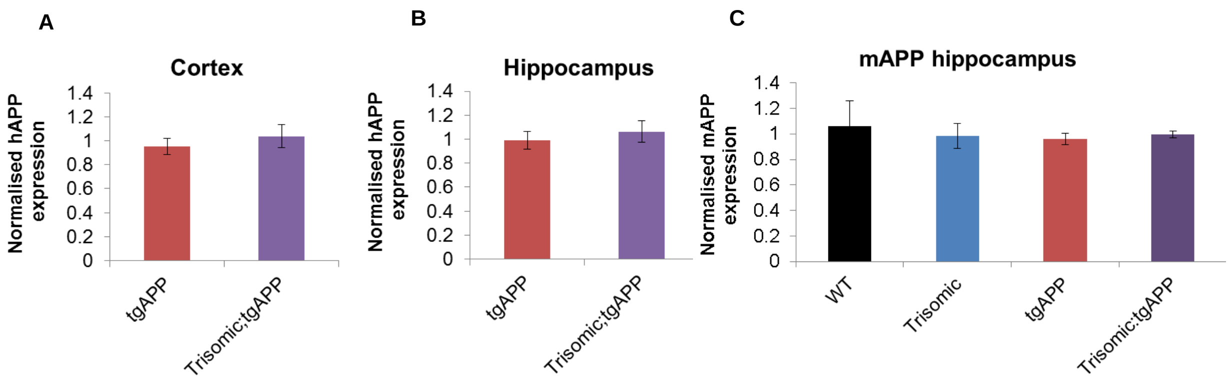

Supplementary Fig. 5: Human and mouse APP mRNA levels in cortex and hippocampus
(**A,B,C**) The amount of human *APP* (carrying the Swedish mutation) and mouse *App* transcript was quantified in total RNA isolated from (**A**) whole cortex or (**B, C**) hippocampus by quantitative reverse-transcription PCR. The geometric mean of the amount of *APP/App* transcript detected relative to *ACTB* and *GAPDH* transcript level was calculated. (**A, B**) Trisomy of chromosome 21 did not significantly alter the amount of human mutant *APP* transcript (cortex T-test p = 0.503, n = 6; hippocampus T-test p = 0.360, n = 6); human mutant *APP* transcript was not detected in wildtype or Tc1 littermates that did not carry tgAPP. (**C**) Trisomy of chromosome 21 (ANOVA F(1,26) = 0.243, p = 0.626) or presence of tg*APP* (ANOVA F(1,26) = 0.727, p = 0.402, trisomy-tgAPP interaction F(1,26) = 0.781, p = 0.676) did not significantly alter the amount of mouse *App* transcript detected. Data show geometric mean ± SEM. Both sexes were studied.

Supplementary Figure 6

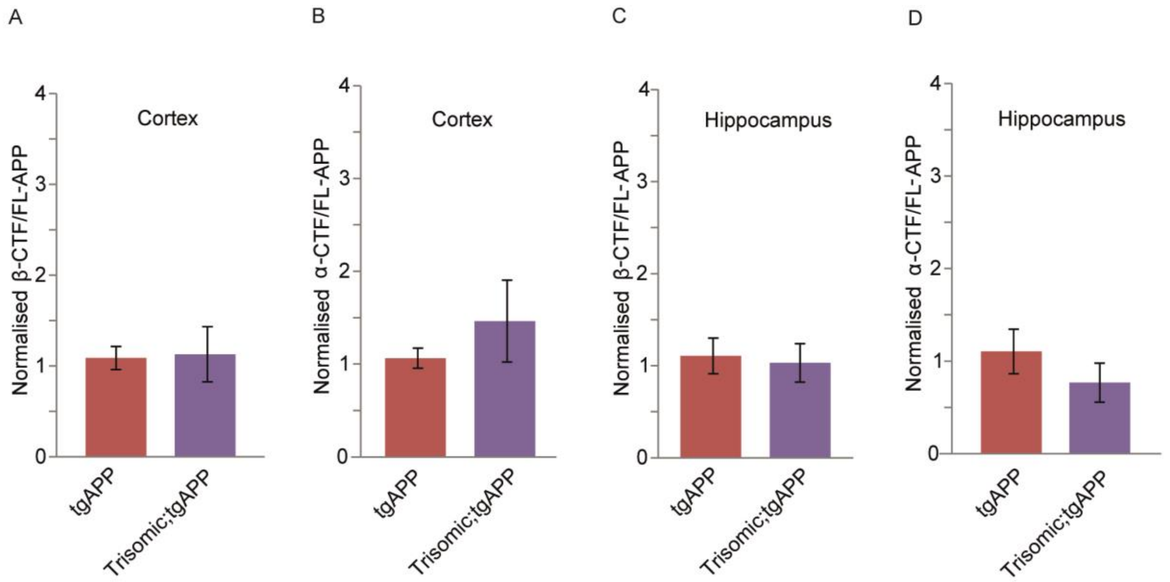

Supplementary Fig. 6: β-CTF/FL-APP and α-CTF/FL-APP in female cortex and hippocampus
(**A-D**) The ratio of β-CTF/FL-APP and α-CTF/FL-APP in (**A,B**) cortex and (**C,D**) hippocampus from female mice was measured by western blot, using anti-APP-C-terminal antibody A8717. (**A,C**) No difference in the β-CTF/FL-APP ratio (T-test, cortex p = 0.903, tgAPP n = 7, Trisomic;tgAPP n = 9; hippocampus p = 0.796, tgAPP n = 10, Trisomic;tgAPP n = 9) or the (**B,D**) α-CTF/FL-APP ratio (T-test, cortex p = 0.396, tgAPP n = 7, Trisomic;tgAPP n = 9, hippocampus p = 0.312, tgAPP n = 10, Trisomic;tgAPP n = 7) was observed between tgAPP and Trisomic;tgAPP females. Data show mean ± SEM.

Supplementary Figure 7

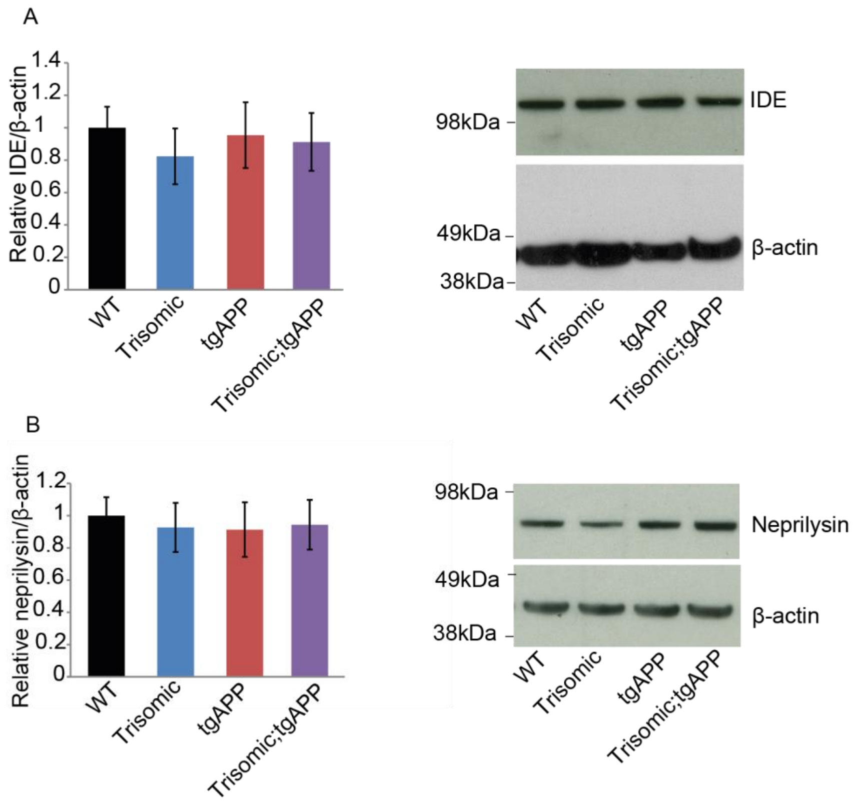

Supplementary Fig. 7: The amount of insulin degrading enzyme and neprilysin in cortex
(**A, B**) The amount of (**A**) insulin degrading enzyme (IDE) and (**B**) neprilysin in total cortical proteins was measured by western blot, an anti-β-actin antibody was used as a control for protein loading. Trisomy did not significantly alter the amount of Aβ clearance enzymes (ANOVA IDE trisomy $F(1,14) = 0.108$, $p = 0.748$, trisomy-tgAPP interaction $F(1,14) = 0.110$, $p = 0.745$, neprilysin trisomy $F(1,23) = 0.041$, $p = 0.841$, trisomy-tgAPP interaction $F(1,23) = 0.076$, $p = 0.786$). Cropped western blots 4 lanes of a 8 lane gel. Data show mean ± SEM. Both sexes were analysed.

Supplementary table 1

Cases of human frontal cortex used for the study of γ-secretase activity

Type	Sex	Age at death	Post mortem delay (hours)
Healthy control	m	54	9
Healthy control	m	53	19
Healthy control	f	59	19
Healthy control	m	65	28
Healthy control	m	55	41
Healthy control	m	46	24
**Control group mean**		**55.3**	**23.3**
AD-DS	f	58	not known
AD-DS	f	45	9
AD-DS	f	48	24
AD-DS	m	63	40
AD-DS	f	58	48
AD-DS	m	55	77
**AD-DS group mean**		**54.5**	**39.6**
